# Needs and supporting tools for primary care physicians to improve care of patients with vertigo and dizziness: a national survey

**DOI:** 10.3389/fneur.2023.1254105

**Published:** 2023-08-29

**Authors:** Georgios Mantokoudis, Andreas Zwergal, Dierik Heg, Hassen Kerkeni, Suzie Diener, Roger Kalla, Athanasia Korda, Claudia Candreia, Antje Welge-Lüssen, Alexander Andrea Tarnutzer

**Affiliations:** ^1^Department of Otorhinolaryngology, Head and Neck Surgery, Bern University Hospital, University of Bern, Bern, Switzerland; ^2^German Center for Vertigo and Balance Disorders (DSGZ), LMU University Hospital, Munich, Germany; ^3^Department of Neurology, LMU University Hospital, Munich, Germany; ^4^CTU Bern, University of Bern, Bern, Switzerland; ^5^Department of Neurology, Bern University Hospital, University of Bern, Bern, Switzerland; ^6^Practice Neurology, St. Gallen Cantonal Hospital, St. Gallen, Switzerland; ^7^Department of Otorhinolaryngology, Head and Neck Surgery, Cantonal Hospital Lucerne, Lucerne, Switzerland; ^8^Department of Otorhinolaryngology, Head and Neck Surgery, University Hospital Basel, Basel, Switzerland; ^9^Neurology, Cantonal Hospital of Baden, Baden, Switzerland; ^10^Faculty of Medicine, University of Zurich, Zurich, Switzerland

**Keywords:** vertigo, dizziness, survey, challenges, limitations, education

## Abstract

**Background:**

The diagnostic workup and treatment decisions for vertigo or dizziness in primary care can be challenging due to the broad range of possible causes and limited time and expertise of physicians. This can lead to delays in treatment and unnecessary tests. We aimed to identify the unmet needs of primary care physicians (PCPs) and strategies to improve care for dizzy patients.

**Materials and methods:**

An online survey was conducted among board-certified PCPs in Switzerland to explore needs in caring for dizzy patients and potential educational approaches.

**Results:**

Based on responses from 152 participating PCPs, satisfaction and confidence were higher in diagnosing (82%) and treating (76%) acute dizziness compared to episodic/chronic cases (63 and 59%, respectively). Younger PCPs had lower diagnostic yield and confidence. Areas for improvement in specialist interactions included communication between physicians (23%/36%; always/often true), shorter waiting times for consultations (19%/40%), more detailed feedback (36%/35%), and consistent patient back referrals (31%/30%). PCPs expressed interest in hands-on courses, workshops, practical guidelines, web-based algorithms, and digital tools such as printed dizzy diaries and apps for follow-up.

**Conclusion:**

Enhanced dialog between PCPs and specialists is crucial to address the most common unmet needs. Reducing waiting times for referrals and providing clear instructions to specialists for triage are essential. The findings from this survey will guide the development of tools to improve the diagnosis and treatment of dizzy patients. Younger PCPs, who face higher diagnostic uncertainty, should be prioritized for educational approaches such as hands-on courses, workshops, and practical recommendations.

## 1. Introduction

Primary care physicians (PCPs) provide the majority of visits for dizziness or vertigo (51.9%) (1) and the consultation prevalence for vertigo/dizziness in primary care practice varies between 1.0 and 15.5% (2). These findings underline the importance of this leading symptom in primary care, which is also prone to misdiagnosis (3). Importantly, the fraction of dizzy patients receiving no specific diagnosis after the PCP’s evaluation varies significantly between studies (range = 0.0–80.2%) (2) and the reported rate of referral to specialists is low, ranging between 14.9% (1), 22% (4) and 47.8% (5). Thus, in a recent systematic review of the literature, it was concluded that health care of patients with vertigo or dizziness in primary care settings is still suboptimal (6). Furthermore, physical therapy referral was the exception in peripheral and central vestibular disorders (0.5%), despite its known efficacy for, e.g., unilateral or bilateral vestibulopathy (7, 8).

Previous work in the field addressed challenges and limitations in the diagnostic workup and treatment of the dizzy patient in primary care. Major challenges include (1) inconsistent patient descriptions of symptom quality, which increases the risk of misdiagnosis when relying solely on symptom quality (9), (2) the broad spectrum of potential diagnoses leading to uncertainty, particularly in cases involving vestibular symptoms where cerebrovascular diseases can be present in a significant percentage of dizziness consultations (3–4%) (10, 11) but are missed in 35% of cases (12) and (3) the lacking referrals to specialists despite unresolved diagnoses (1, 4).

Short evaluation times, limited diagnostic equipment (as, e.g., Frenzel’s goggles or a Snellen chart), lack of colleague exchange (13), and improvement or resolution of symptoms prior to clinical evaluation pose significant limitations in primary care. This makes it challenging to make important decisions regarding treatment, additional diagnostics, specialist referrals, and emergency department referrals. Important decisions including how to treat the patients’ complaints, when to order additional diagnostics, when to refer to a specialist (which specialty to pick) and when to send to the emergency department (ED) are therefore difficult to make. Such delays in diagnosis and treatment may lead to increased healthcare costs and decreased quality of life (14, 15). At the same time, little is known about the PCPs’ perspectives, needs, and attitudes specifically regarding vertigo management and the support they would need for successful vertigo guideline implementation (13).

We therefore sought to investigate the current state of care of the dizzy patient in a highly developed health-care system as established in Switzerland is and how the diagnostic workup and treatment of the dizzy patient could be improved. The primary aim of this study was therefore to (1) gain more knowledge about the current exposure of both primary care physicians and specialists to dizzy patients, (2) to identify limitations and pitfalls in the diagnostic workup and in the interaction between different specialties (generalists, specialists...) and (3) to ask for specific needs of the involved specialties. To achieve these aims, online surveys were designed for both PCPs and specialists. In this publication, we focus on identified unmet needs and potential educational approaches, whereas the current status of care from the view of the PCPs and the specialists’ perspective are addressed in companion papers.

## 2. Materials and methods

### 2.1. Design of the questionnaire

For this survey-based study a structured anonymous online questionnaire (languages: German and French) was designed by the authors (AZ, GM, AT), targeting board-certified PCPs (entitled “general internal medicine”) working in private practice in Switzerland (see appendix for the full questionnaire). Three main sections were defined to address the pre-specified key aims of the study. While the first section was focusing on the current situation in the assessment of the dizzy patient by PCPs, the second section was addressing limitations faced by the PCPs in the diagnostic workup and in the treatment of the dizzy patient. In a third section, potential strategies to improve the standard of care of the dizzy patient and the interaction between generalists and specialists were discussed and the value of different teaching formats were evaluated. At the very beginning of the questionnaire key epidemiologic information were collected including the setting of the PCPs office (location, number of physicians employed), years of professional experience and professional background.

The estimated time needed to fill out the questionnaire was 20–25 min. The questionnaire was available in both German and French language, the translation from German to French was supervised by a native French speaking expert in the field.

### 2.2. Delivery of the questionnaire, identification of suitable participants

For this online-only questionnaire we defined the target sample size to 150 completed surveys. We used Survey Monkey (Momentive Global Inc., San Mateo, CA, United States) for the delivery of the questionnaire to suitable PCPs and for data extraction from completed surveys. The survey was open to all board-certified PCPs working in private practice in Switzerland and was sent to suitable physicians based on a database of interested PCPs run by healthbook.ch. We aimed for a proportional representation of participants from all parts of Switzerland (i.e., target was set to 100 questionnaires from PCPs living / working in the German-speaking part of Switzerland and 50 questionnaires from PCPs located in the French or Italian speaking part of Switzerland, summarized as “Latin part of Switzerland”). Reimbursement for completion of the questionnaire to reflect the amount of time and effort spent was provided to each participant. Calls for participation were sent out 5 times in total to PCPs in the period from January 2022 to February 2022.

### 2.3. Statistical analysis of the questionnaire

First, a descriptive statistical analysis of the questionnaire was performed, focusing on epidemiologic aspects. Second, univariate and multivariate statistical analyzes were run to validate pre-specified hypotheses. Statistical support was provided by the clinical trial unit (CTU) of the Inselspital Bern (Switzerland). A series of scores to reflect key aspects of the diagnostic workup (both history taking and bedside testing) and educational approaches were predefined by the authors (AZ, GM, AT) and were used to correlate with several epidemiologic aspects including years of professional experience, location of PCPs’ office and reported number of dizzy patients evaluated. These scores were graded based on the extent to which the PCPs agreed with a given procedure or the indicated importance of a proposed measure, ranging from 3 points (very important / fully agreed) and 2 points (rather important / partially agreed) to 1 point (rather unimportant / partially disagree) and 0 points (not important at all / disagree at all). All statistical analyzes were performed using Stata version 17.

## 3. Results

We contacted 5,668 PCPs and a total of 152 completed surveys were included. Epidemiologic key aspects have been reported in the companion paper (Zwergal et al. submitted) in detail. In summary, most participants were male (74%), aged 51 years or older and had their offices located in cities (52%) or agglomerations (29%).

### 3.1. Diagnostic limitations in dizzy patients

#### 3.1.1. Missing diagnoses and self-confidence in the diagnostic workup and treatments initiated

**Figure 1:**
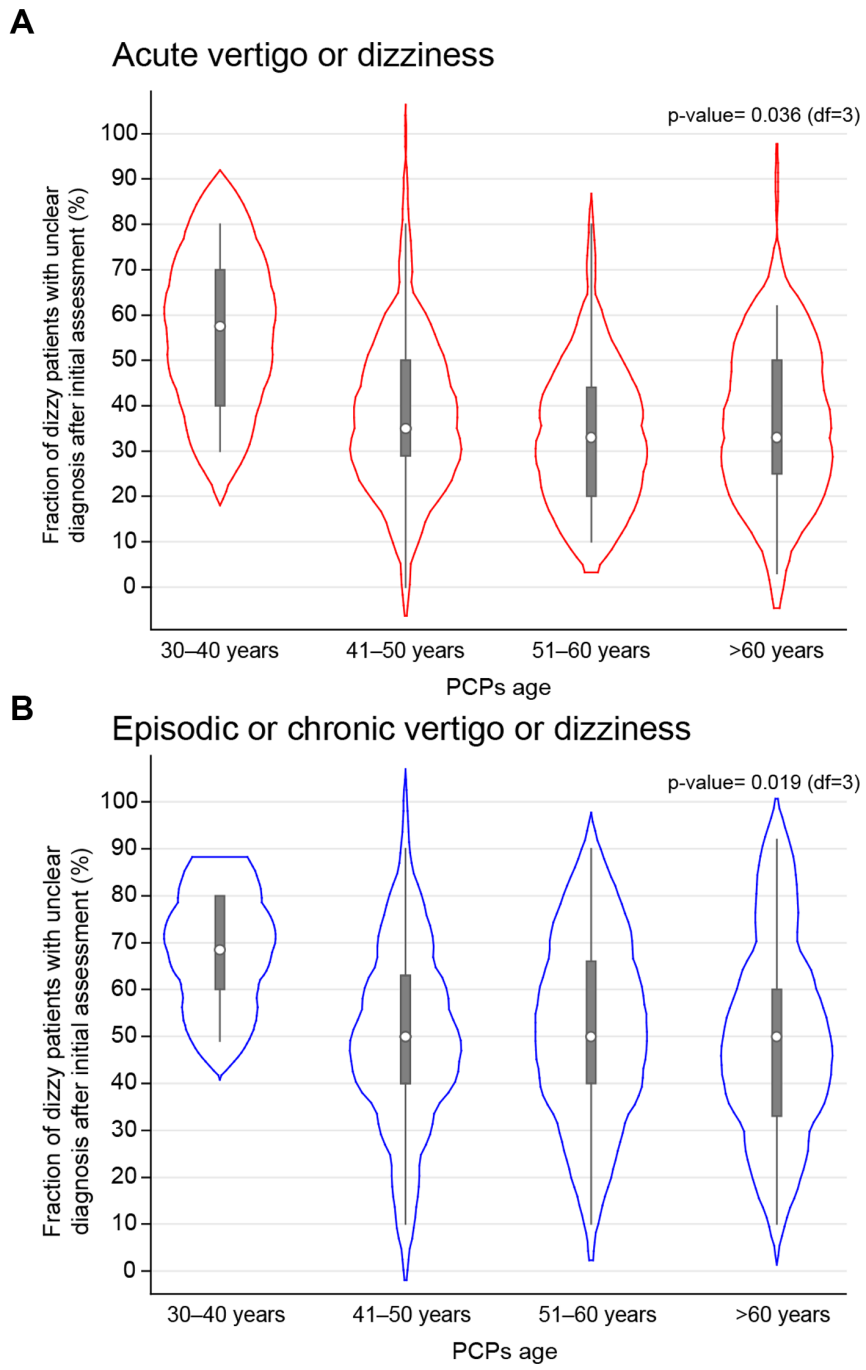
The fraction of dizzy patients which did not receive a specific diagnosis after initial assessment by the PCPs is correlated with the PCPs age, with results shown separately for patients with acute panel **(A)** or episodic/chronic panel **(B)** dizziness/vertigo using a violin plot. All PCPs were assigned to one of four age bins. The white circle represents the median value, the error bars provide the inter-quartile range (with the lower edge of the bar indicating the 25% percentile and the upper end of the bar indicating the 75% percentile and the thin lines indicating the lower and upper adjacent values) and the red panel **(A)** or blue panel **(B)** cloud the distribution of all PCPs of that age group.

As reported previously, PCPs from this survey reported being unable to reach a specific diagnosis after the initial consultation in both acutely dizzy patients [35% (25%; 50%); median and interquartile range (IQR)] and in patients with episodic or chronic dizziness or vertigo [50% (40%; 65.8%)] in a substantial fraction of patients. Noteworthy, more than half of these patients still lacked a specific diagnosis after a completed diagnostic workup. When performing a univariable regression analysis with regards to the odds for lacking a specific diagnosis after the initial assessment using various epidemiologic parameters and several scores (see Supplementary Table S1), only age had a significant impact (*p* = 0.036). Specifically, those PCPs aged 30–40 years demonstrated significantly increased odds {odds ratio (OR) = 2.14, [95%-confidence interval (CI) = 1.16–3.96], *p* = 0.015} for reaching no specific diagnosis in acutely dizzy patients after the first consultation compared to those PCPs aged more than 60 years (see Figure 1A). This was confirmed in a multivariable analysis (*p* = 0.011).

When performing this univariable regression analysis focusing on patients presenting with episodic or chronic dizziness, again a significant effect of age (*p* = 0.019) on the odds of not reaching a specific diagnosis after first assessment was observed (see Supplementary Table S2). Specifically, those PCPs aged 30–40 years demonstrated significantly increased odds [OR = 2.12 (1.28–3.52), *p* = 0.004] for reaching no specific diagnosis in patients with episodic/chronic dizziness/vertigo after the first consultation compared to those PCPs aged more than 60 years (see Figure 1B). This was confirmed in a multivariable analysis (*p* = 0.007).

**Figure 2:**
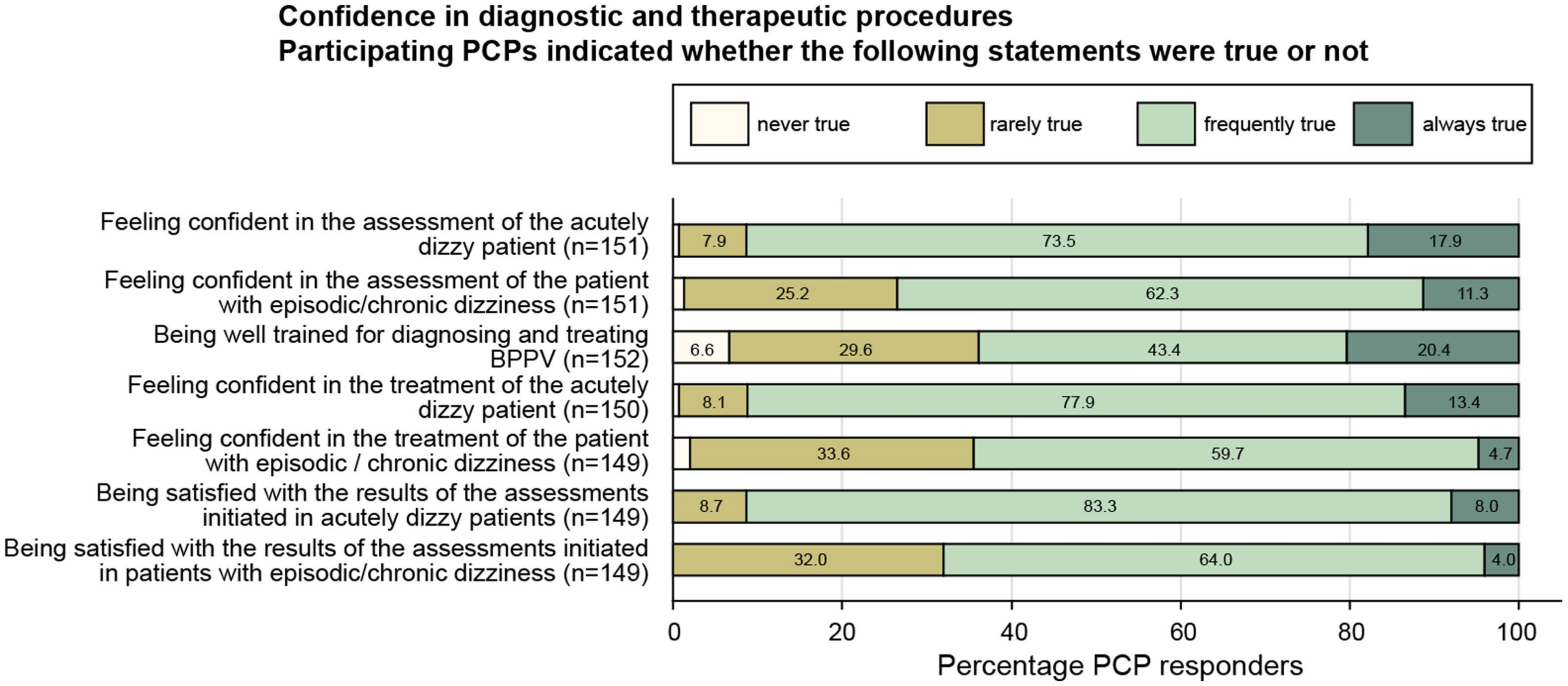
Response patterns of participating PCPs are shown for a series of statements addressing the PCPs satisfaction with diagnostic and therapeutic procedures performed in dizzy patients. For each question, the percentage of PCPs and the extent of agreement they indicated (ranging from “never true” to “always true”) are illustrated. For each question the number (*n*) of valid replies are provided in brackets.

A majority of PCPs indicated that they always or often felt confident in their assessment and treatment of the patient with acute (91%/91%) or episodic / chronic (74%/64%) vertigo or dizziness and that they were always or often satisfied with the results of the diagnostic workup performed (91%/68%; acute / episodic or chronic vertigo/dizziness) (see Figure 2 for details).

#### 3.1.2. Challenges faced when referring patients to specialists

With regards to referrals to specialists for further diagnostic workup and/or treatment, a majority of PCPs agreed that it was always/often true that the waiting time for the assessment of the patient with acute (19%/68%) or episodic / chronic (14%/65%) vertigo or dizziness by the specialist was appropriate (see Supplementary Figure S1 for details). Likewise, a majority of PCPs indicated being satisfied with the assessment performed by the specialist most of the time.

### 3.2. Unmet needs identified by the PCPs and ways to improve the care of the dizzy patient

#### 3.2.1. Interaction between PCPs and specialists

When referring dizzy patients for further assessment to specialists, a majority of participating PCPs agreed that they would like to see an improved communication between the referring PCP and the specialist (23%/36%; always/often true) and that they would like to see shorter waiting times for consults with specialists (19%/40%) (see Supplementary Figure S1 for details). Likewise, PCPs indicated that they would like to receive more precise instructions by the specialists to better understand what information is required from the referring PCP (20%/36%; always true/often true) and that they would like to receive more detailed feedback from the specialist (36%/35%). With regards to follow-up consultations, more PCPs indicated that they expect the specialists to consistently refer patients back to the PCPs for further management (31%/30%; always/often true) than that the specialist takes over follow-up consults of the dizzy patient (12%/33%).

#### 3.2.2. Approaches to improve the PCPs’ knowledge about vertigo and dizziness

**Figure 3:**
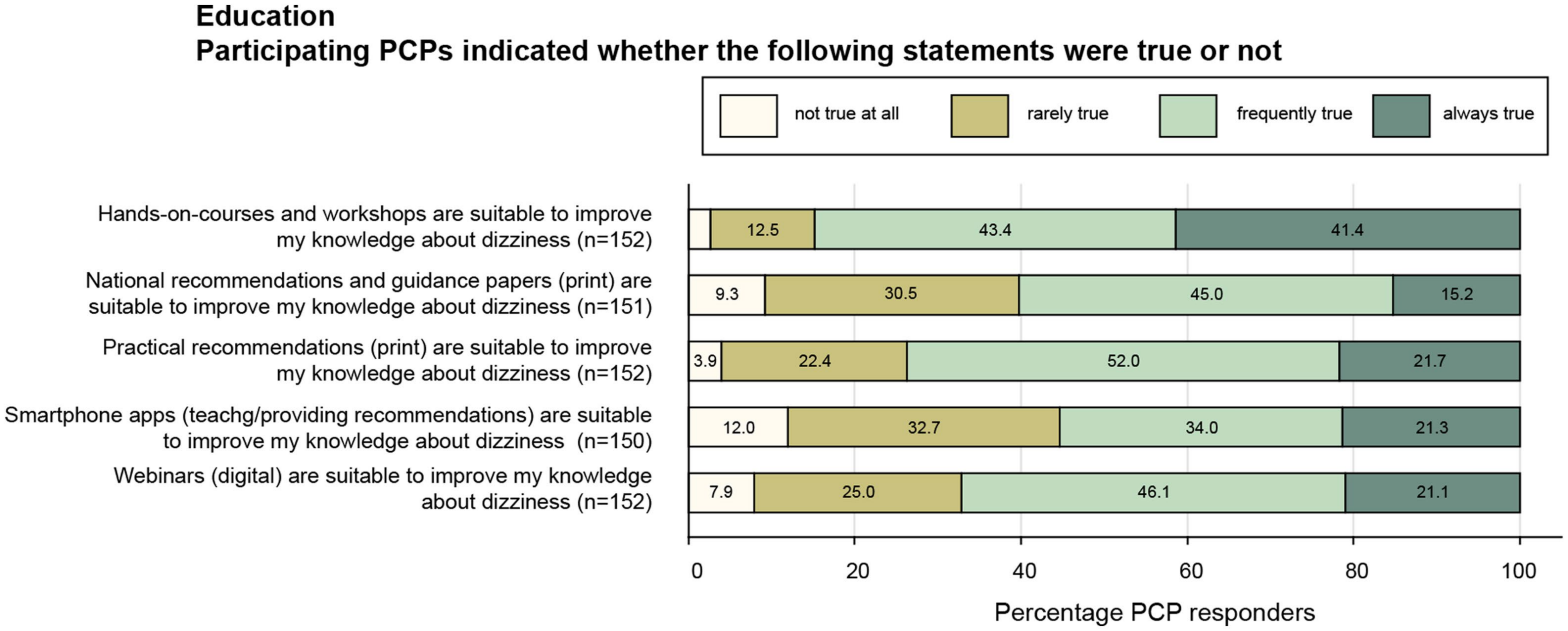
Response patterns of participating PCPs are shown for a series of statements focusing on educational approaches to improve the PCPs knowledge about vertigo and dizziness. For each question, the percentage of PCPs and the extent of agreement they indicated (ranging from “never true” to “always true”) are illustrated. For each question the number (n) of valid replies are provided in brackets.

Among different strategies proposed, participating PCPs considered hands-on-courses and workshops (41%/43%; always/often true) and practical (printed) recommendations (22 52%) most often suitable to improve their knowledge about dizziness (Figure 3). Digital contents such as webinars (21%/46%) and smartphone apps for teaching and providing recommendations (21%/34%) were also considered suitable to improve their skills in taking care of the dizzy patient by a majority of PCPs. This was also true for national recommendations and printed guidance papers, with rates of agreement of 15% (always true) and 45% (often true).

When assessing the calculated score for analog (i.e., print) educational tools (i.e., a score based on the indicated importance of hands-on-courses / workshops, national recommendations on print, practical recommendations on print), no correlation with the PCPs’ age was found (*p* = 0.49). Likewise, there was no correlation between age and a digital educational tools score (i.e., a score based on the indicated importance of smartphone apps and webinars) (*p* = 0.33).

#### 3.2.3. Approaches to improve the care of dizzy patients

Among different digital approaches proposed, participating PCPs most often considered web-based digital pathways / algorithms helpful in the diagnostic workup (70%) and when treating (71%) dizzy patients. Rates for agreement for other digital tools (app-based digital pathways / algorithms and web-portals providing clinical cases) were slightly lower, see Table 1 for details. For following-up on dizzy patients, the use of a printed dizzy diary (59%) and apps including a digital dizzy diary (51%) were considered helpful by the largest fraction of PCPs. With regards to different educational strategies proposed, printed brochures for patients (81%) were considered most helpful by participating PCPs, followed by printed flyers for patients (57%) and app-based digital platforms (55%).

**Table 1:** Tools to improve the management of the dizzy patient.

Tools considered helpful in the diagnosis/treatment	Fractions (%) of agreement (diagnosis/treatment)
Digital pathways/algorithms (web-based)	107/152 (70%)/108/152 (71%)
Digital pathways/algorithms (Smartphone App)	96/152 (63%)/84/152 (55%)
Webportal with clinical cases	87/152 (57%)/93/152 (61%)
Other	3/152 (2%)*/5/152 (3%)†
Tools considered helpful in the follow-up	Fractions (%) of agreement
Dizzy diary (print)	89/152 (59%)
App for follow-up including a dizzy diary (digital)	78/152 (51%)
Web-based follow-up (digital)	69/152 (45%)
Webportal with clinical cases	48/152 (32%)
Other	2/152 (1%)‡
Tools considered helpful for patient education	Fractions (%) of agreement
Brochure for patients (print)	123/152 (81%)
Flyer for patients (print)	87/152 (57%)
Digital platform (app-based)	84/152 (55%)
Dizzy diary (print)	79/152 (52%)
Digital platform (web-based)	68/152 (45%)

*Other approaches considered helpful for improving the diagnostic workup mentioned were discussions in working groups (quality circles, *n* = 1), providing video instructions for provocation/reposition maneuvers in BPPV (*n* = 1), providing such tools as print (*n* = 1). †Other approaches considered helpful for improving the treatment mentioned were providing a suitable text book (*n* = 1), providing such tools as print (*n* = 2) or handouts for PCPs (*n* = 1) or patients (*n* = 1). ‡ Other follow up strategies considered helpful were providing such tools in print (*n* = 2).

## 4. Discussion

This publication examines limitations and pitfalls in the diagnostic workup and interactions between generalists and specialists in the care of dizzy patients. We also focus on the specific needs of PCPs regarding knowledge improvement and patient education/follow-up. By identifying these limitations and proposing tools, we aim to develop strategies to enhance the care of dizzy patients in Switzerland. We also aim to identify valuable sources of support for caregivers and contribute to the development of a national guidance paper on the diagnosis and treatment of dizzy patients.

In general, in patients presenting with episodic or chronic vertigo/dizziness a specific diagnosis was reached less often than in acutely dizzy patients, and consecutively, PCPs were less satisfied in the assessment and treatment of patients with episodic / chronic dizziness. Therefore, it is not surprising, that when interviewing PCPs, one main challenge identified is establishing a definite diagnosis in the dizzy patient (13). With regards to improving the PCPs’ knowledge in handling the dizzy patient, in-person courses and workshops were considered most valuable, whereas digital contents were somewhat less popular. In the following we will critically discuss key findings and limitations, aiming to identify most suitable approaches to overcome current obstacles in the care of the dizzy patient.

### 4.1. Identifying diagnostic limitations and challenges in specialist interactions

We found a discrepancy between the high rate of PCPs indicating that they were always or often satisfied with the results of the diagnostic workup performed (91% / 68%; acute / episodic or chronic vertigo/dizziness) the substantial fraction of undiagnosed patients even after completion of a after specific workup (20% / 31.5%; acute / episodic or chronic vertigo / dizziness). This may indicate that PCPs considered unspecific diagnoses as sufficient to initiate (symptomatic) treatment, resulting in potentially delayed or missed diagnoses and targeted treatment.

In addition, PCPs felt more confident in the diagnostic assessment of acutely dizzy patients than in patients with episodic or chronic dizziness/vertigo. This could be related to the patient presenting to the PCP outside of the actual episodes (i.e., not demonstrating specific oculomotor / vestibular findings) and secondary compensatory mechanisms such as reweighting of sensory input including inappropriate overweighting of specific sensory input, resulting in, e.g., visual dominance. While we hypothesized that being more familiar with key elements of history taking and bedside examination essential for the dizzy patient (aggregated in so-called “superscores,” see companion paper (Zwergal et al., submitted) for detailed description) would result in lower fractions of unclear dizzy cases, regression analyzes performed did not demonstrate such a dependency, neither for patients presenting with acute dizziness nor for those suffering from episodic/chronic dizziness. However, those PCPs aged 30–40 years demonstrated significantly increased odds for reaching no specific diagnosis in both patients with acute [2.14, (1.16–3.96), *p* = 0.015] and episodic/chronic [2.12 (1.28–3.52), *p* = 0.004] dizziness/vertigo after the first consultation compared to those PCPs aged more than 60 years. This finding may be interpreted in different ways. On one hand, it may point to more limited professional experience of younger PCPs and thus failing more often to identify a specific diagnosis. On the other hand, it could also indicate a more rigorous approach and thus higher thresholds to consider a suspected diagnosis confirmed. In either way, this finding underlines that when providing teaching activities to PCPs, age should be taken into account as well.

In the interaction between referring PCPs and involved specialists no major challenges were identified. Important aspects such as the waiting time and the results of the specialized assessment were perceived as often or always satisfactory by at least 75% of PCPs. At the same time, however, 59% of PCPs would like to see shorter waiting times for consults with specialists. Thus, attempts to shorten waiting times should be intensified.

Noteworthy, the PCPs indicated that the fraction of patients being often/always satisfied by the specialist’s assessment was substantially higher in the setting of acute dizziness or vertigo than in episodic/chronic vertigo or dizziness. This is not surprising, since the diagnosis of episodic vertigo is more challenging. Patients with an episodic vertigo such as Menière’s disease or vestibular migraine need at least several episodes in order to fulfill all diagnostic criteria (16, 17). The diagnostic challenge is also reflected by the higher fractions of patients receiving no specific diagnosis after a full assessment.

Detailed feedback from specialists was a top priority for PCPs, with 70% indicating its importance. Additionally, 59% of PCPs highlighted the need to reduce waiting times for specialist consultations and improve dialog between PCPs and specialists. Receiving more detailed instructions what information is needed from the referring PCP was considered important by 56% of PCPs. Overall, there was a preference of participating PCPs that the specialists consistently refer patients back after their assessment for further management. An improved dialog between PCPs and specialists may increase the diagnostic yield of referrals to specialists and reduce waiting times.

### 4.2. Enhancing PCPs’ knowledge of vertigo and dizziness

Based on the PCPs’ feedback on preferred educational approaches, in person courses (84%; hands-on courses, workshops) and printed practical recommendations (74%) should be prioritized, as these formats were considered to be always or at least often helpful most frequently by the PCPs. National recommendations and guidance papers were considered helpful (agreed always or often) by 3 out of 5 PCPs. The lower levels of priority of digital contents such as webinars (agreed always or often in 67%) and smartphone apps (agreed always or often in 55%) may be linked to the demographics of the participating PCPs, with 62% being aged more than 50 years. Noteworthy, in person courses are more time-consuming and expensive than online courses and cannot be easily scaled to larger groups.

### 4.3. Helpful tools in the care of the dizzy patient

Based on the indicated preferences, web-based or app-based digital algorithms and pathways (reaching fractions of agreement of 71 and 55%, respectively) should be prioritized for supporting the PCPs in the diagnosis and treatment of the dizzy patient. Likewise, for following up on dizzy patients, use of a printed dizzy diary was considered useful by the largest fraction of PCPs (59%), followed by a smartphone app (51%) for follow-up (including digital dizzy diary). Thus, these formats should be prioritized. Regarding educational materials for patients, a strong preference for printed information material–especially a brochure for patients–was observed, with an acceptance rate of 81%. In comparison digital (web-based or app-based) contents and other printed materials (flyer, dizzy diary) were considered useful by about half of all PCPs.

In an interview-based semi-structured survey German PCPs were open to both educational meetings and organizational interventions (13). In the same study, the importance of helpful and applicable guidelines in the PCPs’ setting was emphasized. Many guidelines, however, are often perceived as too complex and complicated to use. Importantly, guideline implementation should be supported through educational meetings and organizational change (13). Recent efforts have been made in the development of guidelines for emergency physicians taking care of dizzy patients (18). Such efforts can serve as a model for developing guidelines for PCPs in the future.

Digital tools have been assessed regarding their value in the diagnostic workup of the dizzy patient. In a study involving 610 dizzy patients, the effectiveness of an iPad-based program (medx) in predicting and differentiating the six most common clinical cases of vertigo and dizziness was assessed. With high specificity and negative predictive values (exceeding 82.5%), this system was considered helpful to rule out differential diagnoses and may result in reduced costs (19). However, with sensitivity values as low as 34.7% for vestibular migraine, its use can only be considered complementary. Novel app-based diagnostic algorithms are currently under development for application in general practice, which have proven to have a high sensitivity for detecting vestibular stroke and a high specificity to correctly diagnose the most frequent non-vascular vestibular disorders in the hospital setting of acute vertigo or dizziness (20).

### 4.4. Study limitations

While this online survey-based study offers first-hand in-depth insights into the unmet needs and perceived challenges of PCPs when dealing with the dizzy patient, it faces several limitations. First, there may be a selection bias reflected by a low response rate of 2.68% to the invitation to participate in the survey and a majority of participants being male, aged 51 years or older and having their offices located in cities or agglomerations. Thus, with younger and female PCPs being under-represented in our survey, the findings reported here may only partially reflect how younger PCPs or female PCPs approach the dizzy patient. Second, we collected data on the PCPs’ self-reported diagnostic and therapeutic procedures, which may diverge from the actually executed procedure in a specific patient. Third, replies provided strongly depend on accurate reporting on past performance by the participating PCPs, self-judgments on performance and perceived satisfaction of the patient. These aspects may substantially vary among participating PCPs. Forth, we did not collect any information about the participating PCPs’ curriculum, which might be very variable and may include ear-nose-throat and/or neurology training in some PCPs.

## 5. Conclusion

Patients with episodic or chronic vertigo/dizziness are less likely to receive a specific diagnosis compared to those with acute symptoms, leading to lower satisfaction among PCPs in assessing and treating these patients. Strengthening the interaction between PCPs and specialists is crucial to address current limitations, reduce waiting times, and provide necessary information for triage. Clearly communicating the preferred follow-up strategy (back referral to PCP vs. follow-ups with specialist) will optimize patient flow and reduce unnecessary specialist visits. Improving PCPs’ knowledge of handling patients with episodic/chronic vertigo or dizziness can be achieved through a combination of in-person courses and digital contents. Younger PCPs, who face higher diagnostic uncertainty, should be prioritized for educational approaches such as hands-on courses, workshops, and practical recommendations. The findings from this survey will guide the development of tools to improve the diagnosis and treatment of dizzy patients.

## Data availability statement

The original contributions presented in the study are included in the article/Supplementary material, further inquiries can be directed to the corresponding author.

## Author contributions

GM: Conceptualization, Methodology, Validation, Writing–review and editing. AZ: Conceptualization, Methodology, Validation, Writing–review and editing. DH: Formal analysis, Methodology, Validation, Writing–review and editing. HK: Methodology, Writing–review and editing. SD: Methodology, Writing–review and editing. RK: Methodology, Writing–review and editing. AK: Methodology, Writing–review and editing. CC: Methodology, Writing–review and editing. AW-L: Methodology, Writing–review and editing. AT: Conceptualization, Data curation, Formal analysis, Funding acquisition, Methodology, Supervision, Validation, Writing–original draft, Writing–review and editing.

## Funding

Funding for reimbursement for participation in the survey and for statistical analyzes outsourced to the Inselspital were provided by Schwabe Pharma. GM was funded by the Swiss National Science Foundation #320030_173081.

## Conflict of interest

The authors declare that the research was conducted in the absence of any commercial or financial relationships that could be construed as a potential conflict of interest.

## Publisher’s note

All claims expressed in this article are solely those of the authors and do not necessarily represent those of their affiliated organizations, or those of the publisher, the editors and the reviewers. Any product that may be evaluated in this article, or claim that may be made by its manufacturer, is not guaranteed or endorsed by the publisher.
