# Supplementary material for: Needs and supporting tools for primary care physicians to improve care of patients with vertigo and dizziness: a national survey
Source: Front Neurol. 2023 Aug 29;14:1254105. doi: 10.3389/fneur.2023.1254105 (PMC10495563; doi:10.3389/fneur.2023.1254105)
Supplement: Supplementary file 1 [file Data_Sheet_1.PDF]

Appendix 1 – additional tables / figures from PCP survey

Supplementary figures

Figure S1: PCPs satisfaction with referrals to specialists

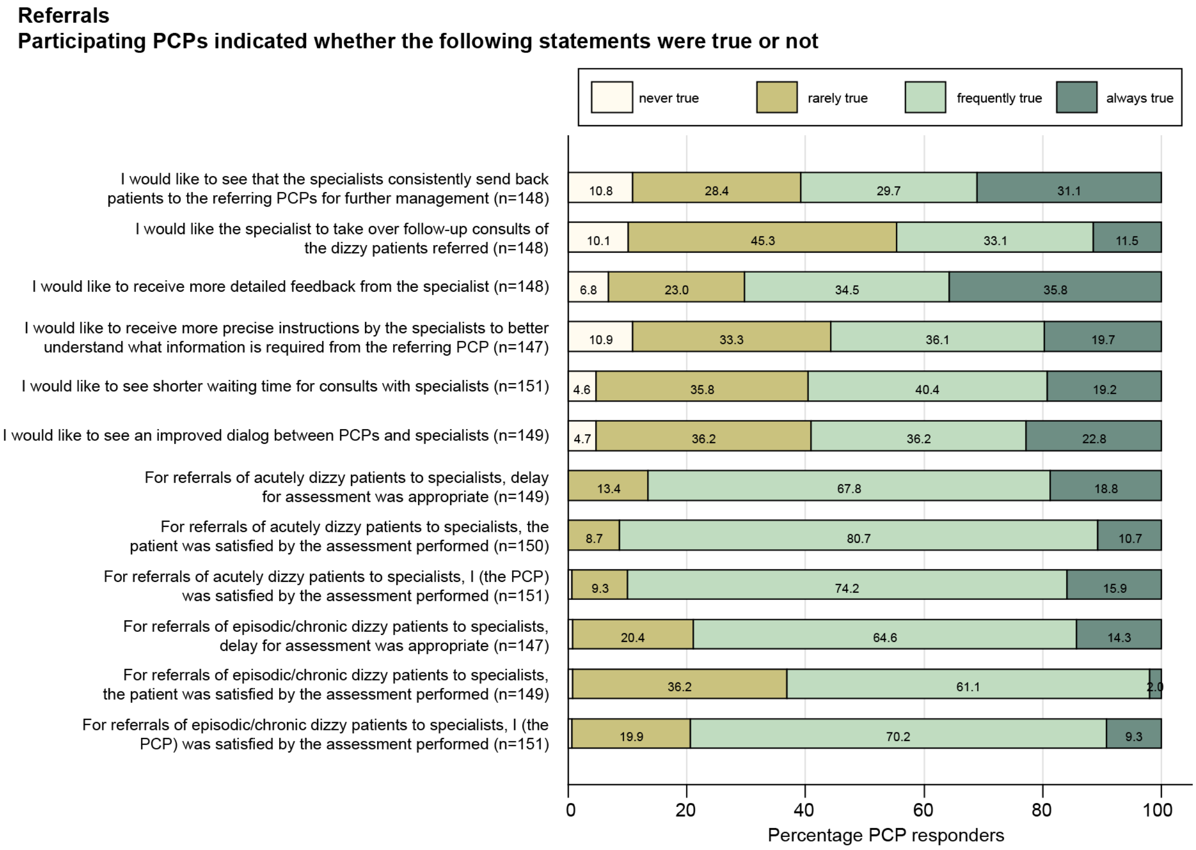

Figure legend:
Response patterns of participating PCPs are shown for a series of statements focusing on the PCPs satisfaction regarding referrals of dizzy patients to specialists. For each question, the percentage of PCPs and the extent of agreement they indicated (ranging from “rarely true” to “always true”) are illustrated. For each question the number (n) of valid replies are provided in brackets.

Tables

Table S1: Predictors for a lacking specific diagnosis in acutely dizzy patients after the first consultation

Table S1: Predictors for a lacking specific diagnosis in acutely dizzy patients after the first consultation			
	**Univariable**		
**Predictor**	**Sample size (n)**	**Odds ratio (95% CI)**	**p-value**
Age			0.036
aged 30-40 years	6	2.14 (1.16-3.96)	0.015
aged 41-50 years	52	1.07 (0.78-1.48)	0.667
aged 51-60 years	55	0.90 (0.66-1.22)	0.479
aged > 60 years	39	[Ref.]	
Gender			
male	112	[Ref.]	
female	40	1.09 (0.82-1.46)	0.545
Years of professional experience	152	0.91 (0.78-1.06)	0.240
Location of PCPs office			
German part of Switzerland	101	[Ref.]	
Latin part of Switzerland	51	0.86 (0.65-1.13)	0.276
Number of dizzy patients seen monthly	152	0.99 (0.98-1.01)	0.292
Availability of Frenzel goggles	152	1.02 (0.80-1.31)	0.849
Score "timing and triggers" (0-100%)	152	1.04 (0.91-1.19)	0.575
Score "ataxia of stance and gait" (0-100%)	152	0.98 (0.90-1.07)	0.685
Score "oculomotor and vestibular signs" (0-100%)	152	0.94 (0.88-1.01)	0.100
Superscore acute dizziness (0-100%)*	152	0.91 (0.79-1.04)	0.163
	**Multivariable**		
**Predictor**	**Sample size (n)**	**Adjusted odds ratio (95% CI)**	**p-value**
Age			0.031
aged 30-40 years	6	2.28 (1.20-4.33)	0.011
aged 41-50 years	52	1.05 (0.77-1.44)	0.743
aged 51-60 years	55	0.89 (0.66-1.21)	0.463
aged > 60 years	39	[Ref.]	
Score "oculomotor and vestibular signs" (0-100%)	152	0.93 (0.84-1.03)	0.169
Superscore acute dizziness (0-100%)*	152	1.01 (0.83-1.24)	0.888

Abbreviations: CI=confidence interval; PCP=primary care provider; Ref=reference

* The "superscore acute dizziness" combines the "essential for acute dizziness" score (assessing gait, Romberg test, head-impulse test, spontaneous nystagmus with fixation and without fixation, testing for gaze-evoked nystagmus, testing for a skew deviation and for hearing loss (using finger rubbing)) and the "timing and triggers" score (asking for the frequency of dizzy episodes, for triggers including specific movements and situations, the duration of dizzy episodes and other, accompanying symptoms)

Table S2: Predictors for a lacking specific diagnosis in patients with episodic/chronic dizziness after the first consultation

**Table S2: Predictors for a lacking specific diagnosis in patients with episodic / chronic dizziness after the first consultation**			
	**Univariable**		
**Predictor**	**Sample size (n)**	**Odds ratio (95% CI)**	**p-value**
Age			0.019
aged 30-40 years	6	2.12 (1.28-3.52)	0.004
aged 41-50 years	52	1.00 (0.72-1.40)	0.993
aged 51-60 years	55	1.07 (0.77-1.48)	0.685
aged > 60 years	39	[Ref.]	
Gender			
male	112	[Ref.]	
female	40	0.94 (0.71-1.27)	0.704
Years of professional experience	152	0.96 (0.82-1.13)	0.623
Location of PCPs office			
German part of Switzerland	101	[Ref.]	
Latin part of Switzerland	51	0.98 (0.75-1.28)	0.884
Number of dizzy patients seen monthly	152	0.99 (0.98-1.00)	0.226
Score "timing and triggers" (0-100%)	152	1.03 (0.90-1.18)	0.676
Score "episodic/chronic dizziness" (0-100%)	152	0.96 (0.86-1.06)	0.404
Superscore episodic/chronic dizziness (0-100%)*	152	0.97 (0.83-1.13)	0.705
	**Multivariable**		
**Predictor**	**Sample size (n)**	**Adjusted odds ratio (95% CI)**	**p-value**
Age			0.046
aged 30-40 years	6	2.13 (1.22-3.71)	0.007
aged 41-50 years	52	1.02 (0.73-1.42)	0.924
aged 51-60 years	55	1.11 (0.80-1.54)	0.542
aged > 60 years	39	[Ref.]	

Abbreviations: CI=confidence interval; PCP=primary care provider; Ref=reference

* The "superscore episodic/chronic dizziness" combines the "essential for episodic/chronic dizziness" score (performing provocation maneuvers, the head-impulse test, gait analysis and the Romberg test) and the "timing and triggers" score (asking for the frequency of dizzy episodes, for triggers including specific movements and situations, the duration of dizzy episodes and other, accompanying symptoms)

Fragebogen für die PrimärversorgerInnen:

3 Abschnitte

1. **Status quo in der Abklärung / Behandlung**
2. **Bestehende Probleme in der Primärversorgung von SchwindelpatientInnen**
3. **Ausblick in die Zukunft – Verbesserungswünsche**

Bitte beachten Sie, dass nur medizinische Fachpersonen dazu befugt sind, diesen Fragebogen auszufüllen. Sofern dies zutrifft und nur, falls Sie den Fragebogen vollständig ausgefüllt haben, wird dies vergütet.

Status quo in der Abklärung / Behandlung

Epidemiologische Eckdaten

* 1. Wie alt sind Sie?

- ◯ <30 Jahre
- ◯ 30–40 Jahre
- ◯ 41–50 Jahre
- ◯ 51–60 Jahre
- ◯ >60 Jahre

* 2. Geschlecht?

- ◯ männlich
- ◯ weiblich
- ◯ keine Angabe

* 3. Wieviele Jahre Berufserfahrung (nach Abschluss Ihres Medizinstudiums) haben Sie?

0 Jahre 40

* 4. Welchen Facharzttitel / welche Facharzttitel besitzen Sie?

- ☐ Allergologie
- ☐ Chirurgie
- ☐ Dermatologie
- ☐ FMH Allgemeine innere Medizin
- ☐ Gastroenterologie
- ☐ Hämatologie
- ☐ Kardiologie
- ☐ Nephrologie
- ☐ Neurochirurgie
- ☐ Neurologie
- ☐ ORL
- ☐ Pädiatrie
- ☐ Pneumologie
- ☐ Psychiatrie
- ☐ Urologie

Anderes

* 5. Wieviele ÄrztInnen arbeiten in Ihrer Praxis?

- Einzelpraxis
- 2–4
- 5–8
- >8

* 6. Wieviele PatientInnen sehen Sie im Schnitt pro Tag?

0 PatientInnen 50

* 7. Wieviel Zeit wenden Sie im Schnitt pro PatientIn auf?

0 Minuten 30

* 8. Bitte beschreiben Sie die Lage Ihrer Praxis?

- Praxis in der Stadt
- Praxis in der Agglomeration
- Praxis auf dem Land

* 9. In welchem Kanton liegt Ihre Praxis?

- Aargau (AG)
- Appenzell Ausserrhoden (AR)
- Appenzell Innerrhoden (AI)
- Basel-Stadt (BS)
- Basel-Landschaft (BL)
- Bern (BE)
- Fribourg / Freiburg (FR)
- Genève / Genf (GE)
- Glarus (GL)
- Graubünden (GR)
- Jura (JU)
- Luzern (LU)
- Neuchâtel / Neuenburg (NE)
- Nidwalden (NW)
- Obwalden (OW)
- St.Gallen (SG)
- Schaffhausen (SH)
- Schwyz (SZ)
- Solothurn (SO)
- Thurgau (TG)
- Ticino / Tessin (TI)
- Uri (UR)
- Valais / Wallis (VS)
- Vaud / Waadt (VD)
- Zug (ZG)
- Zürich (ZH)

Bestehende Probleme in der Primärversorgung von SchwindelpatientInnen

Limitationen in der Abklärung / Behandlung von PatientInnen mit dem Leitsymptom Schwindel

* 10. Wieviele PatientInnen mit dem Leitsymptom Schwindel sehen Sie pro Monat?

0 PatientInnen 100

* 11. Welcher Anteil entfällt dabei auf PatientInnen mit akuten Schwindelsymptomen (erstmalig)?

0 Prozent (%) 100

* 12. Welcher Anteil entfällt dabei auf PatientInnen mit episodischen Schwindelsymptomen (in Attacken auftretend)?

0 Prozent (%) 100

* 13. Welcher Anteil entfällt dabei auf PatientInnen mit chronischem Schwindel (persistierend)?

0 Prozent (%) 100

* 14. Wieviel Zeit wenden Sie im Schnitt pro PatientIn mit Schwindel auf?

- ◯ Weniger Zeit wie für normale PatientInnen
- ◯ Gleich viel Zeit wie für normale PatientInnen
- ◯ Mehr Zeit wie für normale PatientInnen

* 15. Welche Fragen sind für Sie in der Anamneseerhebung bei PatientInnen mit Leitsymptom Schwindel besonders wichtig zur Diagnosestellung?

	trifft gar nicht zu	trifft eher nicht zu	trifft eher zu	trifft bestimmt zu	keine Antwort
Welches ist die Art des aufgetretenen Schwindels (Drehschwindel, Schwankschwindel, Benommenheitsgefühl, Gangunsicherheit)?	○	○	○	○	○
Wie häufig treten die Schwindelattacken auf?	○	○	○	○	○
Wird der Schwindel durch bestimmte Bewegungen ausgelöst?	○	○	○	○	○
Wird der Schwindel in bestimmten Situationen ausgelöst?	○	○	○	○	○
Wie lange dauert eine Schwindelattacke?	○	○	○	○	○
Wie stark ist die Schwindelintensität?	○	○	○	○	○
Liegt Nausea und Erbrechen vor?	○	○	○	○	○
Spürt der Patient eine Falltendenz in eine Richtung?	○	○	○	○	○
Besteht eine Ohrerkrankung (Hörminderung, Tinnitus, Ohrschmerzen)?	○	○	○	○	○
Bestehen weitere Symptome zusammen mit dem Schwindel (Migräne / Spannungskopfschmerzen, neurologische Störungen wie z.B. Dysarthrie oder Dysphagie, andere)?	○	○	○	○	○
Medikamentenanamnese	○	○	○	○	○
Kam es zu einem vorangehenden Trauma (Schädel, HWS)?	○	○	○	○	○

* 16. Wie wichtig sind für Sie die folgenden Untersuchungen bei PatientInnen mit Leitsymptom Schwindel in Ihrer Praxis?

	gar nicht wichtig	eher nicht wichtig	eher wichtig	sehr wichtig	keine Antwort
Gangproben (Strichgang, Blindstrichgang)	◯	◯	◯	◯	◯
Romberg Test	◯	◯	◯	◯	◯
Unterberger Test	◯	◯	◯	◯	◯
Beurteilung hinsichtlich eines Spontannystagmus bei Fixation	◯	◯	◯	◯	◯
Beurteilung hinsichtlich eines Spontannystagmus bei aufgehobener Fixation (z.B. unter der Frenzelbrille)	◯	◯	◯	◯	◯
Kopfimpuls-Test	◯	◯	◯	◯	◯
Beurteilung hinsichtlich eines Blickrichtungsnystagmus	◯	◯	◯	◯	◯
Alternierender Abdecktest (Cover-Test)	◯	◯	◯	◯	◯
Suche nach einer Hörminderung (Fingerreiben, leises Sprechen)	◯	◯	◯	◯	◯
Lagerungsmanöver bei Verdacht auf benignen paroxysmalen Lagerungsschwindel (BPLS)	◯	◯	◯	◯	◯
Allgemeine neurologische Untersuchung (z.B. Lähmungen, Fühlstörungen)	◯	◯	◯	◯	◯
Prüfung der Okulomotorik (Hinweise auf Augenmuskelparesen?)	◯	◯	◯	◯	◯
Otoskopie	◯	◯	◯	◯	◯

* 17. Welche diagnostischen Manöver bei Verdacht auf BPLS kennen Sie?

- ☐ Dix-Hallpike-Manöver
- ☐ Supine-Roll-Manöver / Barbecue 90°
- ☐ Inverses Hallpike-Manöver
- ☐ Bow and Lean Test

* 18. Welche diagnostischen Manöver werden bei Ihnen angewendet?

- [ ] Dix-Hallpike-Manöver
- [ ] Supine-Roll-Manöver / Barbecue 90°
- [ ] Inverses Hallpike-Manöver
- [ ] Bow and Lean Test

* 19. Welche der folgenden Untersuchungsinstrumente stehen Ihnen in Ihrer Praxis zur Verfügung?

- [ ] Frenzelbrille
- [ ] Otoskop
- [ ] Sehtafel
- [ ] Hörtest (inkl. Smartphone-basierte Hörtests)
- [ ] Stimmgabel zur Vibrationstestung (hinsichtlich Polyneuropathie)
- [ ] Keine der oben genannten

* 20. Welchen Anteil der PatientInnen mit akutem Schwindel weisen Sie zum Spezialisten/zur Spezialistin zur weiteren diagnostischen Aufarbeitung weiter?

0 Prozent (%) 100

* 21. Welchen Anteil der PatientInnen mit chronischem / episodischem Schwindel weisen Sie zum Spezialisten/zur Spezialistin zur weiteren diagnostischen Aufarbeitung weiter?

0 Prozent (%) 100

* 22. Welchen SpezialistInnen weisen Sie PatientInnen mit Schwindel am häufigsten weiter? Sortieren Sie bitte die nachfolgenden Antworten durch Anklicken, Ziehen und Ablegen entsprechend.

- Neurologie
- ORL
- Notfall
- interdisziplinäre Schwindelsprechstunde
- Kardiologie
- Psychiatrie
- Neurochirurgie
- Wirbelsäulenchirurgie

* 23. Welche Diagnosen stellen Sie bei Ihren PatientInnen mit Leitsymptom Schwindel am häufigsten? Sortieren Sie bitte die nachfolgenden Antworten durch Anklicken, Ziehen und Ablegen entsprechend (Top 6, N/A: Not Applicable).

- ☐ BPLS (benigner paroxysmaler Lagerungsschwindel) ☐ N/A
- ☐ somatoformer Schwindel (phobischer Schwindel) ☐ N/A
- ☐ vestibuläre Neuritis ☐ N/A
- ☐ Schwindel/Gangunsicherheit bei Polyneuropathie ☐ N/A
- ☐ multifaktorieller Schwindel ☐ N/A
- ☐ unklarer Schwindel ☐ N/A
- ☐ vestibuläre Migräne ☐ N/A
- ☐ Morbus Menière ☐ N/A
- ☐ kardiovaskuläre Ursachen ☐ N/A

* 24. Welche Befunde bei PatientInnen mit akutem Schwindel machen für Sie eine umgehende weiterführende Abklärung erforderlich?

	trifft gar nicht zu	trifft selten zu	trifft häufig zu	trifft immer zu	keine Antwort
Ausgeprägte Gangunsicherheit	○	○	○	○	○
Nausea und Erbrechen	○	○	○	○	○
Begleitende Lähmungen, Sprechstörungen, Fühlstörungen oder Sehstörungen	○	○	○	○	○
Das Vorliegen eines Nystagmus	○	○	○	○	○
Das gleichzeitige Vorliegen einer einseitigen, neu aufgetretenen Hörminderung	○	○	○	○	○
Eine Falltendenz beim freien Sitzen oder selbständigen Stehen, sodass der Patient aufgefangen werden muss.	○	○	○	○	○
Isolierte Kopfschmerzen	○	○	○	○	○
Isolierter Tinnitus	○	○	○	○	○
Erhöhter Blutdruck	○	○	○	○	○

Status quo in der Abklärung / Behandlung: Therapeutische Massnahmen

* 25. Welchem Anteil Ihrer PatientInnen mit Leitsymptom akuter Schwindel verschreiben Sie gezielte Physiotherapie (Gleichgewichtstraining)?

0 Prozent (%) 100

* 26. Welchem Anteil Ihrer PatientInnen mit Leitsymptom chronischer / episodischer Schwindel verschreiben Sie gezielte Physiotherapie (Gleichgewichtstraining)?

0 Prozent (%) 100

* 27. Welchem Anteil Ihrer PatientInnen mit Leitsymptom akuter Schwindel verschreiben Sie Antiemetika?

0 Prozent (%) 100

* 28. Welchem Anteil Ihrer PatientInnen mit Leitsymptom chronischer / episodischer Schwindel verschreiben Sie Antiemetika?

0 Prozent (%) 100

* 29. Welchem Anteil Ihrer PatientInnen mit Leitsymptom akuter Schwindel verschreiben Sie Antivertiginosa?

0 Prozent (%) 100

* 30. Welchem Anteil Ihrer PatientInnen mit Leitsymptom chronischer / episodischer Schwindel verschreiben Sie Antivertiginosa?

0 Prozent (%) 100

* 31. Welche Antivertiginosa verordnen Sie regelmässig?

- [ ] Betahistin
- [ ] Ginkgo biloba Extrakte
- [ ] Kortikosteroide
- [ ] Flunarizin
- [ ] Cinnarizin+Dimenhydrinat

Anderes

* 32. Welche der folgenden Aussagen treffen für Sie bei PatientInnen mit Verdacht auf benignen paroxysmalen Lagerungsschwindel (BPLS) zu?

	trifft gar nicht zu	trifft selten zu	trifft häufig zu	trifft immer zu	keine Antwort
Bei PatientInnen mit Verdacht auf BPLS verschreibe ich Antivertiginosa	○	○	○	○	○
Bei PatientInnen mit Verdacht auf BPLS verschreibe ich Antiemetika	○	○	○	○	○
Bei PatientInnen mit Verdacht auf BPLS führe ich Provokationsmanöver durch	○	○	○	○	○
Bei PatientInnen mit Verdacht auf BPLS gebe ich Instruktionen zur Selbstreposition ab	○	○	○	○	○
Bei PatientInnen mit Verdacht auf BPLS gebe ich eine Broschüre oder Skizze als Instruktionsmaterial zur Selbstreposition ab	○	○	○	○	○
Bei PatientInnen mit Verdacht auf BPLS verweise ich auf Videos im Internet als Instruktionsmaterial zur Selbstreposition	○	○	○	○	○
Bei PatientInnen mit wiederkehrendem BPLS verschreibe ich Vitamin D	○	○	○	○	○

* 33. Welche der folgenden Repositionsmanöver führen Sie bei PatientInnen mit nachgewiesenem BPLS durch?

- [ ] Epley-Manöver
- [ ] Semont-Manöver
- [ ] Gufoni-Manöver
- [ ] Barbecue-Manöver
- [ ] Anderes

* 34. Welche der folgenden Massnahmen führen Sie durch, wenn Sie die Verdachtsdiagnose einer akuten vestibulären Neuritis (d.h. einer akuten Entzündung des Gleichgewichtsnervs) stellen?

	trifft gar nicht zu	trifft selten zu	trifft häufig zu	trifft immer zu	keine Antwort
Weiterverweisung an den SpezialistInnen (Neurologie/ORL)	○	○	○	○	○
Weiterverweisung auf die Notfallstation	○	○	○	○	○
Weiterverweisung an die Radiologie für ein CT-Schädel	○	○	○	○	○
Weiterverweisung an die Radiologie für ein MRI-Schädel	○	○	○	○	○
Behandlung mit Steroiden	○	○	○	○	○
Behandlung mit Virostatika	○	○	○	○	○
Behandlung mit Antiemetika	○	○	○	○	○
Behandlung mit Antivertiginosa	○	○	○	○	○

* 35. Welche der folgenden Massnahmen führen Sie bei PatientInnen mit chronischem / episodischem Schwindel (>3 Monate Dauer) durch?

	trifft gar nicht zu	trifft selten zu	trifft häufig zu	trifft immer zu	keine Antwort
Weiterverweisung an den SpezialistInnen (Neurologie/ORL)	○	○	○	○	○
Weiterverweisung an eine interdisziplinäre Schwindelsprechstunde	○	○	○	○	○
Durchführung von Provokationsmanövern	○	○	○	○	○
Behandlung mit Antivertiginosa	○	○	○	○	○
Behandlung mit Antiemetika	○	○	○	○	○
Behandlung mit Physiotherapie (Fokus Gleichgewichtstraining / Gangschulung)	○	○	○	○	○
Keine, Sie warten ab	○	○	○	○	○

Bestehende Probleme in der Primärversorgung von Schwindelpatienten

Limitationen in der Abklärung / Behandlung von PatientInnen mit dem Leitsymptom Schwindel

* 36. Wie häufig bleibt bei Ihren PatientInnen mit dem Leitsymptom akuter Schwindel die Diagnose unklar nach der Erstkonsultation?

0 Prozent (%) 100

* 37. Wie häufig bleibt bei Ihren PatientInnen mit dem Leitsymptom chronischer / episodischer Schwindel die Diagnose unklar nach der Erstkonsultation?

0 Prozent (%) 100

* 38. Wie häufig bleibt bei Ihren PatientInnen mit dem Leitsymptom akuter Schwindel die Diagnose unklar auch nach zusätzlichen, durch Sie veranlasste Abklärungen?

0 Prozent (%) 100

* 39. Wie häufig bleibt bei Ihren PatientInnen mit dem Leitsymptom chronischer / episodischer Schwindel die Diagnose unklar auch nach zusätzlichen, durch Sie veranlasste Abklärungen?

0 Prozent (%) 100

* 40. Fühlen Sie sich der Abklärung von PatientInnen mit dem Leitsymptom akuter Schwindel gewachsen?

- ◯ trifft gar nicht zu
- ◯ trifft selten zu
- ◯ trifft häufig zu
- ◯ trifft immer zu
- ◯ keine Antwort

* 41. Fühlen Sie sich der Abklärung von PatientInnen mit dem Leitsymptom chronischer / episodischer Schwindel gewachsen?

- ◯ trifft gar nicht zu
- ◯ trifft selten zu
- ◯ trifft häufig zu
- ◯ trifft immer zu
- ◯ keine Antwort

* 42. Sind Sie mit den Ergebnissen der initiierten Abklärung bei PatientInnen mit dem Leitsymptom akuter Schwindel zufrieden?

- trifft gar nicht zu
- trifft selten zu
- trifft häufig zu
- trifft immer zu
- keine Antwort

* 43. Sind Sie mit den Ergebnissen der initiierten Abklärung bei PatientInnen mit dem Leitsymptom chronischer / episodischer Schwindel zufrieden?

- trifft gar nicht zu
- trifft selten zu
- trifft häufig zu
- trifft immer zu
- keine Antwort

* 44. Fühlen Sie sich der Behandlung von PatientInnen mit dem Leitsymptom akuter Schwindel gewachsen?

- trifft gar nicht zu
- trifft selten zu
- trifft häufig zu
- trifft immer zu
- keine Antwort

* 45. Fühlen Sie sich der Behandlung von PatientInnen mit dem Leitsymptom chronischer / episodischer Schwindel gewachsen?

- trifft gar nicht zu
- trifft selten zu
- trifft häufig zu
- trifft immer zu
- keine Antwort

* 46. Wurden Sie im Rahmen Ihrer Ausbildung, Fort- oder Weiterbildung gut ausgebildet, um einen Lagerungsschwindel mittels Manöver zu diagnostizieren und zu behandeln?

- trifft gar nicht zu
- trifft selten zu
- trifft häufig zu
- trifft immer zu
- keine Antwort

* 47. Wenn Sie PatientInnen mit dem Leitsymptom akuter Schwindel an SpezialistInnen zur weiteren Abklärung / Behandlung zuweisen, erfolgt diese Beurteilung angesichts der Dringlichkeit innerhalb eines angemessenen Zeitrahmens?

- trifft gar nicht zu
- trifft selten zu
- trifft häufig zu
- trifft immer zu
- keine Antwort

* 48. Wenn Sie PatientInnen mit dem Leitsymptom chronischer / episodischer Schwindel an SpezialistInnen zur weiteren Abklärung / Behandlung zuweisen, erfolgt diese Beurteilung angesichts der Dringlichkeit innerhalb eines angemessenen Zeitrahmens?

- trifft gar nicht zu
- trifft selten zu
- trifft häufig zu
- trifft immer zu
- keine Antwort

* 49. Wenn Sie PatientInnen mit dem Leitsymptom akuter Schwindel an SpezialistInnen zur weiteren Abklärung / Behandlung zuweisen, erfolgt diese Beurteilung zur Zufriedenheit der PatientInnen?

- trifft gar nicht zu
- trifft selten zu
- trifft häufig zu
- trifft immer zu
- keine Antwort

* 50. Wenn Sie PatientInnen mit dem Leitsymptom chronischer / episodischer Schwindel an SpezialistInnen zur weiteren Abklärung / Behandlung zuweisen, erfolgt diese Beurteilung zur Zufriedenheit der PatientInnen?

- trifft gar nicht zu
- trifft selten zu
- trifft häufig zu
- trifft immer zu
- keine Antwort

* 51. Wenn Sie PatientInnen mit dem Leitsymptom akuter Schwindel an SpezialistInnen zur weiteren Abklärung / Behandlung zuweisen, erfolgt diese Beurteilung zu Ihrer Zufriedenheit?

- trifft gar nicht zu
- trifft selten zu
- trifft häufig zu
- trifft immer zu
- keine Antwort

* 52. Wenn Sie PatientInnen mit dem Leitsymptom chronischer / episodischer Schwindel an SpezialistInnen zur weiteren Abklärung / Behandlung zuweisen, erfolgt diese Beurteilung zu Ihrer Zufriedenheit?

- trifft gar nicht zu
- trifft selten zu
- trifft häufig zu
- trifft immer zu
- keine Antwort

Ausblick in die Zukunft – Verbesserungswünsche

* 53. Was wünschen Sie sich von SpezialistInnen zur Verbesserung der Betreuung von PatientInnen mit dem Leitsymptom Schwindel?

	trifft gar nicht zu	trifft selten zu	trifft häufig zu	trifft immer zu	keine Antwort
Verbesserung des Dialogs zwischen SpezialistInnen und PrimärversorgerInnen					
Kürzere Wartezeiten bei Zuweisung					
Genauere Angaben, welche Daten von den ZuweiserInnen bei Überweisungen geliefert werden müssen					
Eine detailliertere Rückmeldung an die ZuweiserInnen					
Die konsequente Weiterbetreuung der PatientInnen					
Die konsequente Rückverweisung der PatientInnen an die ZuweiserInnen					

* 54. Welche der folgenden Massnahmen sind geeignet, um Ihren Kenntnisstand in Sachen Schwindel als PrimärversorgerIn zu verbessern?

	trifft gar nicht zu	trifft selten zu	trifft häufig zu	trifft immer zu	keine Antwort
Webinare (digital)					
Hands-on Kurse / Workshops (physisch)					
Nationale Empfehlungen, Guidance Paper (Print)					
Praxis-Empfehlungen (Print)					
Smartphone-Apps zur Wissensvermittlung oder als Empfehlungen (digital)					

* 55. Welche der folgenden Tools für PrimärversorgerInnen wären hilfreich bei der Diagnose?

- [ ] Webbasierter diagnostischer Pfad/Algorithmus (digital)
- [ ] App für diagnostischen Pfad/Algorithmus (digital)
- [ ] Webportal mit Krankheitsbildern und Fallbeispielen (digital)
- [ ] Anderes

* 56. Welche der folgenden Tools für PrimärversorgerInnen wären hilfreich bei der Therapie?

- [ ] Webbasierter therapeutischer Pfad (digital)
- [ ] Webportal mit Krankheitsbildern und Fallbeispielen (digital)
- [ ] App für therapeutischen Pfad (digital)

Anderes

* 57. Welche der folgenden Tools für PrimärversorgerInnen wären hilfreich bei der Verlaufskontrolle?

- [ ] Webbasierte Verlaufskontrolle (digital)
- [ ] App zur Verlaufskontrolle mit Schwindeltagebuch (digital)
- [ ] Schwindeltagebuch (print)
- [ ] Webportal mit Krankheitsbildern und Fallbeispielen (digital)

Anderes

* 58. Welche der folgenden Tools für PatientInnen wären hilfreich bei der Patientenedukation & Therapiesteuerung?

- [ ] Webbasierte Plattform (digital)
- [ ] Appbasierte Plattform (digital)
- [ ] Broschüre für PatientInnen (print)
- [ ] Schwindeltagebuch (print)
- [ ] Flyer für PatientInnen (print)

Anderes

Persönliche Daten:

Alle persönlichen Angaben auf dieser Seite dienen ausschliesslich dazu, dem/der Teilnehmer/in die Vergütung für das Ausfüllen des Fragebogens zukommen zu lassen. Alle persönlichen Angaben werden vertraulich behandelt und werden weder an Drittpersonen weitergegeben, gespeichert, noch zu statistischen Zwecken genutzt.

* 59. Persönliche Daten

Titel	
Vorname	
Nachname	
Strasse / Nummer	
PLZ / Ort	
Bankverbindung	
GLN-Nummer	

60. Optionale Angaben

Spital	
Abteilung	

* 61. Ich bestätige hiermit, medizinische Fachperson zu sein.

☐ Ja

Questionnaire aux médecins de premier recours :

3 sections

1. **Statu quo dans la clarification / le traitement**
2. **Problèmes existants dans la prise en charge primaire de patient/e/s atteint/e/s de vertiges**
3. **Perspectives – Désirs d'amélioration**

Veuillez noter que seuls les professionnels de la santé sont habilités à remplir ce questionnaire. Si c'est le cas, et seulement si vous avez rempli le questionnaire dans son entier, cela vous sera remboursé.

Statu quo dans la clarification / le traitement

Statu quo – Données épidémiologiques clés

* 1. Quel âge avez-vous ?

- ◯ <30 ans
- ◯ 30–40 ans
- ◯ 41–50 ans
- ◯ 51–60 ans
- ◯ >60 ans

* 2. Sexe ?

- ◯ masculin
- ◯ féminin
- ◯ sans objet

* 3. De combien d'années d'expérience professionnelle (suite à l'achèvement de vos études de médecine) disposez-vous ?

0 ans 40

* 4. De quel titre de médecin spécialiste êtes-vous titulaire ?

- ☐ Allergologie
- ☐ Chirurgie
- ☐ Dermatologie
- ☐ FMH Médecine interne générale
- ☐ Gastroentérologie
- ☐ Hématologie
- ☐ Cardiologie
- ☐ Néphrologie
- ☐ Neurochirurgie
- ☐ Neurologie
- ☐ ORL
- ☐ Pédiatrie
- ☐ Pneumologie
- ☐ Psychiatrie
- ☐ Urologie

Autre

* 5. Combien de praticien/ne/s travaillent au sein de votre cabinet ?

- ◯ Cabinet individuel
- ◯ 2–4
- ◯ 5–8
- ◯ >8

* 6. Combien de patient/e/s voyez-vous en moyenne par jour ?

0 patient/e/s 50

* 7. Combien de temps passez-vous en moyenne par patient/e ?

0 minutes 30

* 8. Veuillez décrire la localisation de votre cabinet :

- ◯ Cabinet en ville
- ◯ Cabinet en agglomération
- ◯ Cabinet à la campagne

* 9. Dans quel canton se trouve votre cabinet ?

- ◯ Argovie
- ◯ Appenzell Rhodes-Extérieures
- ◯ Appenzell Rhodes-Intérieures
- ◯ Bâle-Ville
- ◯ Bâle-Campagne
- ◯ Berne
- ◯ Fribourg
- ◯ Genève
- ◯ Glaris
- ◯ Grisons
- ◯ Jura
- ◯ Lucerne
- ◯ Neuchâtel
- ◯ Nidwald
- ◯ Obwald
- ◯ Saint-Gall
- ◯ Schaffhouse
- ◯ Schwyz
- ◯ Soleure
- ◯ Thurgovie
- ◯ Tessin
- ◯ Uri
- ◯ Valais
- ◯ Vaud
- ◯ Zoug
- ◯ Zurich

Statu quo dans la clarification / le traitement

Prise en charge actuelle de patient/e/s atteint/e/s de vertiges

* 10. Combien de patient/e/s se plaignant principalement de vertiges voyez-vous par mois ?

0 patient/e/s 100

* 11. Quelle est la proportion de patient/e/s atteint/e/s de symptômes vertigineux aigus (survenant pour la première fois) ?

0 pour cent (%) 100

* 12. Quelle est la proportion de patient/e/s atteint/e/s de symptômes vertigineux épisodiques (survenant sous forme de crises) ?

0 pour cent (%) 100

* 13. Quelle est la proportion de patient/e/s atteint/e/s de vertiges chroniques (persistants) ?

0 pour cent (%) 100

* 14. Combien de temps passez-vous en moyenne par patient/e atteint/e de vertiges ?

- Moins de temps qu'avec un/e patient/e normal/e
- Autant de temps qu'avec un/e patient/e normal/e
- Plus de temps qu'avec un/e patient/e normal/e

* 15. Quelles questions revêtent pour vous une importance toute particulière pour la pose du diagnostic lors de l'anamnèse de patient/e/s se plaignant principalement de vertiges ?

	pas du tout importante	plutôt pas importante	plutôt importante	très importante	aucune réponse
Quelle forme prennent les vertiges (vertiges rotatoires, oscillatoires, étourdissements, démarche mal assurée) ?	○	○	○	○	○
À quelle fréquence les crises de vertiges se produisent-elles ?	○	○	○	○	○

Les vertiges sont-ils déclenchés par certains mouvements ?	○	○	○	○	○
Les vertiges sont-ils provoqués par certaines situations ?	○	○	○	○	○
Combien de temps dure une crise de vertiges ?	○	○	○	○	○
Quelle est l’intensité des vertiges ?	○	○	○	○	○
Les vertiges s’accompagnent-ils de nausées ou de vomissements ?	○	○	○	○	○
Le patient ressent-il une tendance à la chute dans un sens donné ?	○	○	○	○	○
Le patient présente-t-il une maladie des oreilles ? (Perte auditive, acouphène, douleurs aux oreilles)	○	○	○	○	○
Les vertiges s’accompagnent-ils d’autres symptômes (migraines / céphalées tensionnelles, troubles neurologiques, comme une dysarthrie ou une dysphagie, autres) ?	○	○	○	○	○
Anamnèse médicamenteuse	○	○	○	○	○
Ces vertiges sont-ils précédés d’un traumatisme (crânien, rachidien) ?	○	○	○	○	○

* 16. Quelle importance revêtent à votre avis les examens suivants auprès de patient/e/s se plaignant principalement de vertiges au sein de votre cabinet ?

	pas du tout important	plutôt pas important	plutôt important	très important	aucune réponse
Tests de marche (marche sur une ligne, marche sur une ligne à l'aveugle)	◯	◯	◯	◯	◯
Signe de Romberg	◯	◯	◯	◯	◯
Épreuve d'Unterberger	◯	◯	◯	◯	◯
Évaluation de la présence d'un nystagmus spontané en cas de fixation	◯	◯	◯	◯	◯
Évaluation de la présence d'un nystagmus spontané en cas de fixation suspendue (par ex. avec les lunettes de Frenzel)	◯	◯	◯	◯	◯
Test de Halmagyi	◯	◯	◯	◯	◯
Évaluation de la présence d'un nystagmus du regard	◯	◯	◯	◯	◯
Test sous écran alterné	◯	◯	◯	◯	◯
Recherche d'une perte auditive (frottement des doigts, chuchotement)	◯	◯	◯	◯	◯
Manœuvre de positionnement en cas de soupçon de vertiges positionnels paroxystiques bénins (VPPB)	◯	◯	◯	◯	◯
Examen neurologique général (par ex. paralysies, troubles de la sensibilité)	◯	◯	◯	◯	◯
Contrôle de l'oculomotricité (signes de paralysie de muscles oculaires ?)	◯	◯	◯	◯	◯
Otoscopie	◯	◯	◯	◯	◯

* 17. Quelles manœuvres diagnostiques connaissez-vous en cas de soupçon de VPPB ?

- [ ] Manœuvre de Dix-Hallpike
- [ ] Manœuvre de roulement sur le dos / Lempert à 90°
- [ ] Manœuvre de Hallpike inversée
- [ ] Test de Choung

* 18. Quelles manœuvres diagnostiques sont exécutées au sein de votre cabinet ?

- [ ] Manœuvre de Dix-Hallpike
- [ ] Manœuvre de roulement sur le dos / Lempert à 90°
- [ ] Manœuvre de Hallpike inversée
- [ ] Test de Choung

* 19. Parmi les instruments d'examen suivants, desquels disposez-vous au sein de votre cabinet ?

- [ ] Lunettes de Frenzel
- [ ] Otoscope
- [ ] Tableau optométrique
- [ ] Test auditif (y compris tests auditifs disponibles sur smartphone)
- [ ] Diapason pour le test de vibration (en cas de polyneuropathie)
- [ ] Aucun des instruments cités

* 20. Quelle proportion de patient/e/s atteint/e/s de vertiges aigus réorientez-vous auprès d'un/e spécialiste en vue d'un approfondissement du diagnostic ?

0 pour cent (%) 100

* 21. Quelle proportion de patient/e/s atteint/e/s de vertiges chroniques/épisodiques réorientez-vous auprès d'un/e spécialiste en vue d'un approfondissement du diagnostic ?

0 pour cent (%) 100

* 22. Auprès de quel/le/s spécialistes réorientez-vous le plus souvent les patient/e/s atteint/e/s de vertiges? Veuillez classer les réponses suivantes en les déplaçant après les avoir activées.

- ☐ Neurologie
- ☐ ORL
- ☐ urgences
- ☐ consultation interdisciplinaire en vertiges
- ☐ cardiologie
- ☐ psychiatrie
- ☐ neurochirurgie
- ☐ chirurgie du rachis

* 23. Quel diagnostic posez-vous le plus fréquemment chez vos patient/e/s se plaignant principalement de vertiges ? Veuillez classer les réponses suivantes en les déplaçant après les avoir activées.

- ☐ VPPB (vertiges positionnels paroxystiques bénins) ☐ N/A
- ☐ vertiges somatoformes (vertiges phobiques) ☐ N/A
- ☐ névrite vestibulaire ☐ N/A
- ☐ vertiges/démarche mal assurée associé/e/s à une polyneuropathie ☐ N/A
- ☐ vertiges multifactoriels ☐ N/A
- ☐ vertiges d’origine incertaine ☐ N/A
- ☐ migraines vestibulaires ☐ N/A
- ☐ maladie de Menière ☐ N/A
- ☐ causes cardiovasculaires ☐ N/A

* 24. Quelles observations auprès de patient/e/s atteint/e/s de vertiges aigus nécessitent immédiatement un examen approfondi ?

	pas du tout	rarement	souvent	toujours	aucune réponse
Altération marquée de la démarche	○	○	○	○	○
Nausée et vomissements	○	○	○	○	○
Paralysies, troubles de l'élocution, de la sensibilité ou troubles visuels concomitants	○	○	○	○	○
Présence d'un nystagmus	○	○	○	○	○
Survenue simultanée d'une nouvelle perte auditive unilatérale	○	○	○	○	○
Tendance à la chute en étant assis/e librement ou en posture debout sans aide, de telle sorte que le patient/la patiente doive être rattrapé/e.	○	○	○	○	○
Céphalées isolées	○	○	○	○	○
Acouphène isolé	○	○	○	○	○
Pression artérielle élevée	○	○	○	○	○

Statu quo dans la clarification / le traitement

Mesures thérapeutiques

* 25. À quelle proportion de vos patient/e/s se plaignant principalement de vertiges aigus prescrivez-vous des séances ciblées de kinésithérapie (entraînement de l'équilibre) ?

0 pour cent (%) 100

* 26. À quelle proportion de vos patient/e/s se plaignant principalement de vertiges chroniques / épisodiques prescrivez-vous des séances ciblées de kinésithérapie (entraînement de l'équilibre) ?

0 pour cent (%) 100

* 27. À quelle proportion de vos patient/e/s se plaignant principalement de vertiges aigus prescrivez-vous des anti-émétiques ?

0 pour cent (%) 100

* 28. À quelle proportion de vos patient/e/s se plaignant principalement de vertiges chroniques / épisodiques prescrivez-vous des anti-émétiques ?

0 pour cent (%) 100

* 29. À quelle proportion de vos patient/e/s se plaignant principalement de vertiges aigus prescrivez-vous des anti-vertigineux ?

0 pour cent (%) 100

* 30. À quelle proportion de vos patient/e/s se plaignant principalement de vertiges chroniques / épisodiques prescrivez-vous des anti-vertigineux ?

0 pour cent (%) 100

* 31. Quels anti-vertigineux prescrivez-vous régulièrement ?

- [ ] Bétahistine
- [ ] Extraits de gingko biloba
- [ ] Corticostéroïdes
- [ ] Flunarizine
- [ ] Cinnarizine+diménhydrinate

Autre

[ ]

* 32. Laquelle/Lesquelles des déclarations suivantes vous correspond/ent dans la prise en charge de patient/e/s chez lesquel/le/s vous soupçonnez des vertiges positionnels paroxystiques bénins (VPPB) ?

	pas du tout	rarement	souvent	toujours	aucune réponse
Aux patient/e/s chez lesquel/le/s je soupçonne des VPPB, je prescris des anti-vertigineux	○	○	○	○	○
Aux patient/e/s chez lesquel/le/s je soupçonne des VPPB, je prescris des anti-émétiques	○	○	○	○	○
Aux patient/e/s chez lesquel/le/s je soupçonne des VPPB, je procède à des manœuvres de provocation	○	○	○	○	○
Aux patient/e/s chez lesquel/le/s je soupçonne des VPPB, je fournis des instructions d’auto-repositionnement	○	○	○	○	○
Aux patient/e/s chez lesquel/le/s je soupçonne des VPPB, je remets une brochure ou un croquis servant de support d’instructions pour l’auto-repositionnement	○	○	○	○	○
Aux patient/e/s chez lesquel/le/s je soupçonne des VPPB, je conseille de consulter des vidéos sur Internet pour s’instruire à l’auto-repositionnement	○	○	○	○	○
Aux patient/e/s présentant des VPPB récurrents, je prescris de la vitamine D	○	○	○	○	○

* 33. Laquelle des manœuvres de repositionnement suivantes réalisez-vous auprès des patient/e/s présentant de manière démontrée des VPPB ?

- [ ] Manœuvre d’Epley
- [ ] Manœuvre de Semont
- [ ] Manœuvre de Gufoni
- [ ] Manœuvre de Lempert
- [ ] Autre

* 34. Laquelle des mesures suivantes exécutez-vous lors de la pose d’un diagnostic présomptif d’une névrite vestibulaire aiguë (c’est-à-dire d’une inflammation aiguë du nerf de l’équilibre) ?

	ne convient pas du tout	convient rarement	convient souvent	convient toujours	aucune réponse
Renvoi à un/e spécialiste (neurologie/ORL)	○	○	○	○	○
Renvoi au service des urgences	○	○	○	○	○
Renvoi au service de radiologie pour une TDM crânienne	○	○	○	○	○
Renvoi au service de radiologie pour un IRM crânien	○	○	○	○	○
Traitement par des stéroïdes	○	○	○	○	○
Traitement par des virostatiques	○	○	○	○	○
Traitement par des anti-émétiques	○	○	○	○	○
Traitement par des antivertigineux	○	○	○	○	○

* 35. Lesquelles des mesures suivantes exécutez-vous auprès de patient/e/s atteint/e/s de vertiges chroniques / épisodiques (pendant >3 mois) ?

	ne convient pas du tout	convient rarement	convient souvent	convient toujours	aucune réponse
Renvoi à un/e spécialiste (neurologie/ORL)	○	○	○	○	○
Renvoi auprès d’une consultation interdisciplinaire en vertiges	○	○	○	○	○
Exécution de manœuvres de provocation	○	○	○	○	○
Traitement par des antivertigineux	○	○	○	○	○
Traitement par des anti-émétiques	○	○	○	○	○
Traitement avec kinésithérapie (concentration : entraînement de l’équilibre / la démarche)	○	○	○	○	○
Aucune, vous attendez	○	○	○	○	○

Problèmes existants dans la prise en charge primaire de patient/e/s atteint/e/s de vertiges

Limites dans la clarification / le traitement de patient/e/s se plaignant principalement de vertiges

* 36. À quelle fréquence le diagnostic de vos patient/e/s se plaignant principalement de vertiges aigus reste-t-il incertain suite à la première consultation ?

0 pour cent (%) 100

* 37. À quelle fréquence le diagnostic de vos patient/e/s se plaignant principalement de vertiges chroniques / épisodiques reste-t-il incertain suite à la première consultation ?

0 pour cent (%) 100

* 38. À quelle fréquence le diagnostic de vos patient/e/s se plaignant principalement de vertiges aigus reste-t-il incertain même après des examens supplémentaires réalisés à votre initiative ?

0 pour cent (%) 100

* 39. À quelle fréquence le diagnostic de vos patient/e/s se plaignant principalement de vertiges chroniques / épisodiques reste-t-il incertain même après des examens supplémentaires réalisés à votre initiative ?

0 pour cent (%) 100

* 40. Vous sentez-vous à même d'examiner des patient/e/s se plaignant principalement de vertiges aigus ?

- pas du tout
- rarement
- souvent
- toujours
- aucune réponse

* 41. Vous sentez-vous à même d’examiner des patient/e/s se plaignant principalement de vertiges chroniques / épisodiques ?

- pas du tout
- rarement
- souvent
- toujours
- aucune réponse

* 42. Êtes-vous satisfait/e des résultats de l’examen initié auprès des patient/e/s se plaignant principalement de vertiges aigus ?

- pas du tout
- rarement
- souvent
- toujours
- aucune réponse

* 43. Êtes-vous satisfait/e des résultats de l’examen initié auprès des patient/e/s se plaignant principalement de vertiges chroniques/épisodiques ?

- pas du tout
- rarement
- souvent
- toujours
- aucune réponse

* 44. Vous sentez-vous à même de traiter des patient/e/s se plaignant principalement de vertiges aigus ?

- pas du tout
- rarement
- souvent
- toujours
- aucune réponse

* 45. Vous sentez-vous à même de traiter des patient/e/s se plaignant principalement de vertiges chroniques / épisodiques ?

- pas du tout
- rarement
- souvent
- toujours
- aucune réponse

* 46. Avez-vous été correctement formé/e dans le cadre de votre instruction, de votre perfectionnement ou de votre formation continue pour pouvoir diagnostiquer et traiter des vertiges positionnels par le biais de manœuvres ?

- pas du tout
- rarement
- souvent
- toujours
- aucune réponse

* 47. Lorsque vous réorientez des patient/e/s se plaignant principalement de vertiges aigus auprès de spécialistes en vue de l’approfondissement de leur examen / traitement, cette évaluation a-t-elle lieu en temps voulu au vu de l’urgence du cas ?

- pas du tout
- rarement
- souvent
- toujours
- aucune réponse

* 48. Lorsque vous réorientez des patient/e/s se plaignant principalement de vertiges chroniques / épisodiques auprès de spécialistes en vue de l’approfondissement de leur examen / traitement, cette évaluation a-t-elle lieu en temps voulu au vu de l’urgence du cas ?

- pas du tout
- rarement
- souvent
- toujours
- aucune réponse

* 49. Lorsque vous réorientez des patient/e/s se plaignant principalement de vertiges aigus auprès de spécialistes en vue de l’approfondissement de leur examen / traitement, cette évaluation a-t-elle lieu à la satisfaction des patient/e/s ?

- pas du tout
- rarement
- souvent
- toujours
- aucune réponse

* 50. Lorsque vous réorientez des patient/e/s se plaignant principalement de vertiges chroniques / épisodiques auprès de spécialistes en vue de l'approfondissement de leur examen / traitement, cette évaluation a-t-elle lieu à la satisfaction des patient/e/s ?

- pas du tout
- rarement
- souvent
- toujours
- aucune réponse

* 51. Lorsque vous réorientez des patient/e/s se plaignant principalement de vertiges aigus auprès de spécialistes en vue de l'approfondissement de leur examen / traitement, cette évaluation a-t-elle lieu à votre satisfaction ?

- pas du tout
- rarement
- souvent
- toujours
- aucune réponse

* 52. Lorsque vous réorientez des patient/e/s se plaignant principalement de vertiges chroniques / épisodiques auprès de spécialistes en vue de l'approfondissement de leur examen / traitement, cette évaluation a-t-elle lieu à votre satisfaction ?

- pas du tout
- rarement
- souvent
- toujours
- aucune réponse

Perspectives – Désirs d’amélioration

Perspectives (désirs / besoins)

* 53. Que désirez-vous de la part des spécialistes en vue d’améliorer la prise en charge de patient/e/s se plaignant principalement de vertiges ?

	ne convient pas du tout	convient rarement	convient souvent	convient toujours	aucune réponse
Amélioration du dialogue entre les spécialistes et les médecins de premiers recours	○	○	○	○	○
Temps d’attente abrégés en cas de réorientation	○	○	○	○	○
Indication précise des données à fournir par les médecins référents lors des réorientations	○	○	○	○	○
Un retour détaillé aux médecins référents	○	○	○	○	○
La poursuite systématique de la prise en charge des patient/e/s	○	○	○	○	○
Le renvoi systématique des patient/e/s aux médecins référents	○	○	○	○	○

* 54. Lesquelles des mesures suivantes conviendraient afin d'améliorer vos connaissances à propos des vertiges en qualité de médecin de premier recours ?

	ne convient pas du tout	convient rarement	convient souvent	convient toujours	aucune réponse
Webinaires (format numérique)	○	○	○	○	○
Cours pratiques / ateliers (en présentiel)	○	○	○	○	○
Recommandations nationales, document d'orientation (support imprimé)	○	○	○	○	○
Recommandations aux cabinets (support imprimé)	○	○	○	○	○
Applications de smartphone destinées à la circulation du savoir ou des recommandations (format numérique)	○	○	○	○	○

* 55. Lesquels des outils suivants adressés aux médecins de premier recours pourraient s'avérer utile dans le cadre du diagnostic ?

- ☐ Parcours/algorithme diagnostique en ligne (format numérique)
- ☐ Application de parcours/algorithme diagnostique (format numérique)
- ☐ Portail en ligne avec tableaux cliniques et exemples de cas (format numérique)
- ☐ Autre

* 56. Lesquels des outils suivants adressés aux médecins de premier recours pourraient s'avérer utiles dans le cadre du traitement ?

- ☐ Parcours thérapeutique en ligne (format numérique)
- ☐ Portail en ligne avec tableaux cliniques et exemples de cas (format numérique)
- ☐ Application de suivi du parcours thérapeutique (format numérique)

Autre

* 57. Lesquels des outils suivants adressés aux médecins de premier recours pourraient s’avérer utiles dans le cadre du suivi ?

- [ ] Suivi en ligne (format numérique)
- [ ] Application de suivi avec journal des vertiges (format numérique)
- [ ] Journal des vertiges (support imprimé)
- [ ] Portail en ligne avec tableaux cliniques et exemples de cas (format numérique)

Autre

* 58. Lesquels des outils suivants adressés aux patient/e/s pourraient s’avérer utiles dans la sensibilisation des patient/e/s et l’aménagement du traitement ?

- [ ] Plate-forme en ligne (format numérique)
- [ ] Plate-forme sur application (format numérique)
- [ ] Brochure aux patient/e/s (support imprimé)
- [ ] Journal des vertiges (support imprimé)
- [ ] Dépliant pour patient/e/s (support imprimé)

Autre

Données Personnelles :

Toutes les données personnelles figurant sur cette page servent exclusivement à faire parvenir au participant la rémunération pour avoir rempli le questionnaire. Toutes les données personnelles sont traitées de manière confidentielle et ne sont ni transmises à des tiers, ni enregistrées, ni utilisées à des fins statistiques.

* 59. Données personnelles :

Titre	
Prénom	
Nom de famille	
Rue / numéro	
NPA / Lieu	
Coordonnées bancaires	
Numéro GLN	

60. Données facultatives

Hôpital	
Service	

* 61. Je confirme par la présente être un professionnel de la santé.

☐ Oui

Primary Care Provider Questionnaire:

3 sections

1. **Status Quo Regarding Diagnosis/Treatment**
2. **Current Problems in the Treatment of Patients with Dizziness**
3. **Prospects for the Future—Desired Improvements**

Please note that only health care professionals are authorized to complete this questionnaire. Compensation will only be provided if this condition applies and if you have completed the questionnaire in full.

Status Quo Regarding Diagnosis/Treatment

Key Epidemiological Data

* 1. How old are you?

- ○ <30 years
- ○ 30–40 years
- ○ 41–50 years
- ○ 51–60 years
- ○ >60 years

* 2. What is your gender?

- ○ male
- ○ female
- ○ not specified

* 3. How many years of professional experience do you have (since completing your medical training)?

0 years 40

* 4. Which medical specialties are you certified in?

- ☐ Allergies
- ☐ Surgery
- ☐ Dermatology
- ☐ FMH [Swiss Medical Association]-certified general internal medicine
- ☐ Gastroenterology
- ☐ Hematology
- ☐ Cardiology
- ☐ Nephrology
- ☐ Neurosurgery
- ☐ Neurology
- ☐ ENT
- ☐ Pediatrics
- ☐ Pulmonology
- ☐ Psychiatry
- ☐ Urology
- ☐ Other

* 5. How many physicians work in your practice?

- ○ Solo practice
- ○ 2–4
- ○ 5–8
- ○ >8

* 6. On average, how many patients do you see per day?

0 patients 50

* 7. On average, how much time do you spend with each patient?

0 minutes 30

* 8. Please describe the location of your practice

- ○ City practice
- ○ Practice is in a greater metropolitan area
- ○ Country practice

* 9. In which Canton of Switzerland is your practice located?

- ○ Aargau (AG)
- ○ Appenzell Ausserrhoden (AR)
- ○ Appenzell Innerrhoden (AI)
- ○ Basel-City (BS)
- ○ Basel-Country (BL)
- ○ Bern (BE)
- ○ Freiburg (FR)
- ○ Geneva (GE)
- ○ Glarus (GL)
- ○ Graubünden (GR)
- ○ Jura (JU)
- ○ Lucerne (LU)
- ○ Neuchâtel (NE)
- ○ Nidwalden (NW)
- ○ Obwalden (OW)
- ○ St. Gallen (SG)
- ○ Schaffhausen (SH)
- ○ Schwyz (SZ)
- ○ Solothurn (SO)
- ○ Thurgau (TG)
- ○ Ticino (TI)
- ○ Uri (UR)
- ○ Valais (VS)
- ○ Vaud (VD)
- ○ Zug (ZG)
- ○ Zurich (ZH)

Current Problems in the Primary Treatment of Patients with Dizziness

Limitations in Diagnosing/Treating Patients Presenting with Dizziness as a Cardinal Symptom

* 10. How many patients with a cardinal symptom of dizziness do you see per month?

0 patients 100

* 11. Of these, what proportion are patients with acute (first-time) dizziness symptoms?

0 percent (%) 100

* 12. Of these, what proportion are patients with episodic dizziness symptoms (in the form of attacks)?

0 percent (%) 100

* 13. Of these, what proportion are patients with chronic (persistent) dizziness?

0 percent (%) 100

* 14. On average, how much time do you spend on each patient with dizziness?

○ Less time than for normal patients
○ Same time as for normal patients
○ More time than for normal patients

* 15. What questions do you consider particularly important in diagnostic terms when taking the history of patients presenting with dizziness as a cardinal symptom?

	Not applicable at all	Somewhat inapplicable	Somewhat applicable	Definitely applicable	No answer
What type of dizziness occurred (vertigo, feeling off-balance, lightheadedness, unsteady gait)?	◯	◯	◯	◯	◯
How often do the attacks of dizziness occur?	◯	◯	◯	◯	◯
Is the dizziness triggered by certain movements?	◯	◯	◯	◯	◯
Is the dizziness triggered in certain situations?	◯	◯	◯	◯	◯
How long does an attack of dizziness last?	◯	◯	◯	◯	◯
How intense is the dizziness?	◯	◯	◯	◯	◯
Is the dizziness accompanied by nausea and vomiting?	◯	◯	◯	◯	◯
Does the patient feel a tendency to fall in one direction?	◯	◯	◯	◯	◯
Are there any ear disorders present (hearing loss, tinnitus, ear pain)?	◯	◯	◯	◯	◯
Are there any other symptoms accompanying the dizziness (migraine/tension headaches, neurological disorders such as dysarthria or dysphagia, others)?	◯	◯	◯	◯	◯
Prior/current medication history	◯	◯	◯	◯	◯
Did the patient experience trauma beforehand (cranial, cervical spine)?	◯	◯	◯	◯	◯

* 16. How important do you consider the following tests to be for patients presenting with dizziness as a cardinal symptom in your practice?

	Not important at all	Somewhat unimportant	Somewhat important	Very important	No answer
Gait tests (tandem gait test, tandem gait test with eyes closed)	○	○	○	○	○
Romberg test	○	○	○	○	○
Unterberger's test	○	○	○	○	○
Assessment for spontaneous nystagmus with fixation	○	○	○	○	○
Assessment for spontaneous nystagmus with suppressed fixation (e.g. using Frenzel goggles)	○	○	○	○	○
Head impulse test	○	○	○	○	○
Assessment for gaze-evoked nystagmus	○	○	○	○	○
Alternate cover test	○	○	○	○	○
Search for hearing loss (rubbed fingers, quiet speech)	○	○	○	○	○
Positional maneuvers for suspected benign paroxysmal positional vertigo (BPPV)	○	○	○	○	○
General neurological examination (e.g. paralysis, dysesthesia)	○	○	○	○	○
Checking ocular motor function (evidence of ocular palsy?)	○	○	○	○	○
Otoscopy	○	○	○	○	○

* 17. Which diagnostic maneuvers for suspected BPPV are you familiar with?

- ☐ Dix-Hallpike maneuver
- ☐ Supine roll test/barbecue 90°
- ☐ Inverse Hallpike maneuver
- ☐ Bow and lean test

* 18. Which diagnostic maneuvers are used in your practice?

- ☐
- ☐ Dix-Hallpike maneuver
- ☐ Supine roll test/barbecue 90°
- ☐ Inverse Hallpike maneuver
- ☐ Bow and lean test

* 19. Which of the following testing instruments are available in your practice for you to use?

- ☐ Frenzel goggles
- ☐ Otoscope
- ☐ Eye chart
- ☐ Hearing test (including smartphone-based hearing tests)
- ☐ Tuning fork for vibration testing (for polyneuropathy)
- ☐ None of the above

* 20. What proportion of patients with acute dizziness do you refer to a specialist in another discipline for further diagnostic workup?

0 percent (%) 100

* 21. What proportion of patients with chronic/episodic dizziness do you refer to a specialist in another discipline for further diagnostic workup?

0 percent (%) 100

* 22. To which specialists do you most often refer patients with dizziness? Please sort the following answers by clicking, dragging, and dropping them in the right order.

- ≣ ▭ Neurology
- ≣ ▭ ENT
- ≣ ▭ Emergency
- ≣ ▭ Interdisciplinary dizziness clinic
- ≣ ▭ Cardiology
- ≣ ▭ Psychiatry
- ≣ ▭ Neurosurgery
- ≣ ▭ Spinal surgery

* 23. What are your most common diagnoses for patients presenting with dizziness as a cardinal symptom? Please sort the following answers by clicking, dragging, and dropping them in the right order (top 6, N/A: not applicable).

- ≣ ▭ BPPV (benign paroxysmal positional vertigo) ☐ N/A
- ≣ ▭ Somatoform dizziness (phobic vertigo) ☐ N/A
- ≣ ▭ Vestibular neuritis ☐ N/A
- ≣ ▭ Dizziness/unsteady gait associated with polyneuropathy ☐ N/A
- ≣ ▭ Multifactorial dizziness ☐ N/A
- ≣ ▭ Dizziness of unknown etiology ☐ N/A
- ≣ ▭ Vestibular migraine ☐ N/A
- ≣ ▭ Meniere’s disease ☐ N/A
- ≣ ▭ Cardiovascular causes ☐ N/A

* 24. In your opinion, which findings in patients with acute dizziness require immediate further investigation/diagnostic workup?

	Not applicable at all	Rarely applicable	Frequently applicable	Always applicable	No answer
Markedly unsteady gait	◯	◯	◯	◯	◯
Nausea and vomiting	◯	◯	◯	◯	◯
Concomitant paralysis, dysarthria, dysesthesia, or vision problems	◯	◯	◯	◯	◯
Presence of nystagmus	◯	◯	◯	◯	◯
Accompanying unilateral, newly occurring hearing loss	◯	◯	◯	◯	◯
A tendency to fall when sitting unsupported or standing unassisted, requiring the patient to be caught.	◯	◯	◯	◯	◯
Isolated headaches	◯	◯	◯	◯	◯
Isolated tinnitus	◯	◯	◯	◯	◯
Elevated blood pressure	◯	◯	◯	◯	◯

Status Quo regarding Diagnosis/Treatment: Therapeutic Measures

* 25. To what proportion of your patients presenting with acute dizziness as a cardinal symptom do you prescribe targeted physical therapy (balance training)?

0 percent (%) 100

* 26. To what proportion of your patients presenting with chronic/episodic dizziness as a cardinal symptom do you prescribe targeted physical therapy (balance training)?

0 percent (%) 100

* 27. To what proportion of your patients presenting with acute dizziness as a cardinal symptom do you prescribe antiemetics?

0 percent (%) 100

* 28. To what proportion of your patients presenting with chronic/episodic dizziness as a cardinal symptom do you prescribe antiemetics?

0 percent (%) 100

* 29. To what proportion of your patients presenting with acute dizziness as a cardinal symptom do you prescribe antivertigo medications?

0 percent (%) 100

* 30. To what proportion of your patients presenting with chronic/episodic dizziness as a cardinal symptom do you prescribe antivertigo medications?

0 percent (%) 100

* 31. Which antivertigo medications do you regularly prescribe?

- ☐ Betahistine
- ☐ Ginkgo biloba extracts
- ☐ Corticosteroids
- ☐ Flunarizine
- ☐ Cinnarizine + dimenhydrinate
- ☐ Other

* 32. In your opinion, which of the following statements apply for patients with suspected benign paroxysmal positional vertigo (BPPV)?

	Not applicable at all	Rarely applicable	Frequently applicable	Always applicable	No answer
I prescribe antivertigo medications for patients with suspected BPPV	○	○	○	○	○
I prescribe antiemetics for patients with suspected BPPV	○	○	○	○	○
I perform provocation maneuvers for patients with suspected BPPV	○	○	○	○	○
I provide self-repositioning instructions to patients with suspected BPPV	○	○	○	○	○
I provide a brochure or outline as self-repositioning instructional material to patients with suspected BPPV	○	○	○	○	○
I provide patients with suspected BPPV with links to online videos as self-repositioning instructional material	○	○	○	○	○
I prescribe vitamin D to patients with recurrent BPPV	○	○	○	○	○

* 33. Which of the following repositioning maneuvers do you perform with patients who have confirmed BPPV?

- ☐
- ☐ Epley maneuver
- ☐ Semont maneuver
- ☐ Gufoni maneuver
- ☐ Barbecue maneuver
- ☐ Other

* 34. Which of the following steps do you take if you diagnose a patient with suspected acute vestibular neuritis (i.e. acute inflammation of the vestibular nerve)?

	Not applicable at all	Rarely applicable	Frequently applicable	Always applicable	No answer
Referral to the relevant specialist (neurology/ENT)	◯	◯	◯	◯	◯
Referral to the Emergency Room	◯	◯	◯	◯	◯
Referral to Radiology for a cranial CT	◯	◯	◯	◯	◯
Referral to Radiology for a cranial MRI	◯	◯	◯	◯	◯
Treatment with steroids	◯	◯	◯	◯	◯
Treatment with virostatics	◯	◯	◯	◯	◯
Treatment with antiemetics	◯	◯	◯	◯	◯
Treatment with antivertigo medications	◯	◯	◯	◯	◯

* 35. Which of the following steps do you take for patients with chronic/episodic dizziness (lasting >3 months)?

	Not applicable at all	Rarely applicable	Frequently applicable	Always applicable	No answer
Referral to the relevant specialist (neurology/ENT)	○	○	○	○	○
Referral to an interdisciplinary dizziness clinic	○	○	○	○	○
Performance of provocation maneuvers	○	○	○	○	○
Treatment with antivertigo medications	○	○	○	○	○
Treatment with antiemetics	○	○	○	○	○
Treatment with physical therapy (focused on balance training/gait training)	○	○	○	○	○
None, you wait and see	○	○	○	○	○

Current Problems in the Treatment of Patients with Dizziness

Limitations in Diagnosing/Treating Patients Presenting with Dizziness as a Cardinal Symptom

* 36. For your patients presenting with acute dizziness as a cardinal symptom, how often does the diagnosis remain unclear after the initial consultation?

0 percent (%) 100

* 37. For your patients presenting with chronic/episodic dizziness as a cardinal symptom, how often does the diagnosis remain unclear after the initial consultation?

0 percent (%) 100

* 38. For your patients presenting with acute dizziness as a cardinal symptom, how often does the diagnosis remain unclear even after further diagnostic workup arranged by you?

0 percent (%) 100

* 39. For your patients presenting with chronic/episodic dizziness as a cardinal symptom, how often does the diagnosis remain unclear even after further diagnostic workup arranged by you?

0 percent (%) 100

* 40. Do you feel equipped to perform the diagnostic workup for patients presenting with acute dizziness as a cardinal symptom?

○ Not applicable at all
○ Rarely applicable
○ Frequently applicable
○ Always applicable
○ No answer

* 41. Do you feel equipped to perform the diagnostic workup for patients presenting with chronic/episodic dizziness as a cardinal symptom?

○
○ Not applicable at all
○ Rarely applicable
○ Frequently applicable
○ Always applicable
○ No answer

* 42. Are you satisfied with the results of the diagnostic workup initiated for patients presenting with acute dizziness as a cardinal symptom?

○ Not applicable at all
○ Rarely applicable
○ Frequently applicable
○ Always applicable
○ No answer

* 43. Are you satisfied with the results of the diagnostic workup initiated for patients presenting with chronic/episodic dizziness as a cardinal symptom?

○ Not applicable at all
○ Rarely applicable
○ Frequently applicable
○ Always applicable
○ No answer

* 44. Do you feel equipped to treat patients presenting with acute dizziness as a cardinal symptom?

○ Not applicable at all
○ Rarely applicable
○ Frequently applicable
○ Always applicable
○ No answer

* 45. Do you feel equipped to treat patients presenting with chronic/episodic dizziness as a cardinal symptom?

○ Not applicable at all
○ Rarely applicable
○ Frequently applicable
○ Always applicable
○ No answer

* 46. During your initial training and in-service training or continuous medical education, were you well trained in how to use maneuvers to diagnose and treat positional vertigo?

○ Not applicable at all
○ Rarely applicable
○ Frequently applicable
○ Always applicable
○ No answer

* 47. If you refer patients presenting with acute dizziness as a cardinal symptom to specialists for further diagnostic workup/treatment, is this assessment performed within a reasonable time frame given the urgency?

- ○ Not applicable at all
- ○ Rarely applicable
- ○ Frequently applicable
- ○ Always applicable
- ○ No answer

* 48. If you refer patients presenting with chronic/episodic dizziness as a cardinal symptom to specialists for further diagnostic workup/treatment, is this assessment performed within a reasonable time frame given the urgency?

- ○ Not applicable at all
- ○ Rarely applicable
- ○ Frequently applicable
- ○ Always applicable
- ○ No answer

* 49. If you refer patients presenting with acute dizziness as a cardinal symptom to specialists for further diagnostic workup/treatment, is this assessment performed to the satisfaction of the patients?

- ○ Not applicable at all
- ○ Rarely applicable
- ○ Frequently applicable
- ○ Always applicable
- ○ No answer

* 50. If you refer patients presenting with chronic/episodic dizziness as a cardinal symptom to specialists for further diagnostic workup/treatment, is this assessment performed to the satisfaction of the patients?

- ○ Not applicable at all
- ○ Rarely applicable
- ○ Frequently applicable
- ○ Always applicable
- ○ No answer

* 51. If you refer patients presenting with acute dizziness as a cardinal symptom to specialists for further diagnostic workup/treatment, is this assessment performed to your satisfaction?

- ○ Not applicable at all
- ○ Rarely applicable
- ○ Frequently applicable
- ○ Always applicable
- ○ No answer

* 52. If you refer patients presenting with chronic/episodic dizziness as a cardinal symptom to specialists for further diagnostic workup/treatment, is this assessment performed to your satisfaction?

- ○ Not applicable at all
- ○ Rarely applicable
- ○ Frequently applicable
- ○ Always applicable
- ○ No answer

Prospects for the Future—Desired Improvements

* 53. What would you like to see from specialists to improve the care of patients presenting with dizziness as a cardinal symptom?

	Not applicable at all	Rarely applicable	Frequently applicable	Always applicable	No answer
Improvement of the dialog between specialists and primary care providers	○	○	○	○	○
Shorter wait times for referrals	○	○	○	○	○
More specific information regarding what data the referring physician needs to provide when referring patients	○	○	○	○	○
More detailed reporting back to referring physicians	○	○	○	○	○
Consistent further care provided to patients	○	○	○	○	○
Consistent referral of patients back to referring physicians	○	○	○	○	○

* 54. Which of the following are appropriate steps to help you improve your familiarity with issues related to dizziness as a primary care provider?

	Not applicable at all	Rarely applicable	Frequently applicable	Always applicable	No answer
Webinars (digital)	◯	◯	◯	◯	◯
Hands-on courses/workshops (in-person)	◯	◯	◯	◯	◯
National recommendations, guidance paper (print)	◯	◯	◯	◯	◯
Practice recommendations (print)	◯	◯	◯	◯	◯
Smartphone apps to convey information or for recommendations (digital)	◯	◯	◯	◯	◯

* 55. Which of the following tools for primary care providers would be helpful for diagnosis?

- ☐ Web-based diagnostic pathway/algorithm (digital)
- ☐ App for diagnostic pathway/algorithm (digital)
- ☐ Web-portal with clinical pictures and sample case reports (digital)
- ☐ Other

* 56. Which of the following tools for primary care providers would be helpful for treatment?

- ☐ Web-based therapeutic pathway (digital)
- ☐ Web-portal with clinical pictures and sample case reports (digital)
- ☐ App for therapeutic pathway (digital)
- ☐ Other

* 57. Which of the following tools for primary care providers would be helpful for follow-up?

- ☐ Web-based follow-up (digital)
- ☐ App for follow-up with dizziness diary (digital)
- ☐ Dizziness diary (print)
- ☐ Web-portal with clinical pictures and sample case reports (digital)
- ☐ Other

* 58. Which of the following tools for patients would be helpful for patient education & treatment management?

- ☐ Web-based platform (digital)
- ☐ App-based platform (digital)
- ☐ Brochure for patients (print)
- ☐ Dizziness diary (print)
- ☐ Flyer for patients (print)
- ☐ Other

Personal Data:

All personal information on this site is solely for the purpose of providing the participant with compensation for completing the questionnaire. All personal information will be treated in confidence and will neither be divulged to third parties, nor saved, nor used for statistical purposes.

* 59. Personal data

Title	
First name	
Last name	
Street/number	
ZIP code/City	
Bank details	
GLN number	

60. Optional information

Hospital	
Department	

* 61. I hereby confirm that I am a health care professional.

☐ Yes
